# Transmission of deformed wing virus between *Varroa destructor* foundresses, mite offspring and infested honey bees

**DOI:** 10.1186/s13071-022-05463-9

**Published:** 2022-09-23

**Authors:** Vincent Piou, Frank Schurr, Eric Dubois, Angélique Vétillard

**Affiliations:** 1 Laboratoire Evolution Et Diversité Biologique, UMR5174, CNRS-Université de Toulouse III-IRD, INU Jean-François Champollion-Université Paul Sabatier, 31062 Toulouse, France; 2 Unit of Honey Bee Pathology, Sophia Antipolis Laboratory, French Agency for Food, Environment and Occupational Health & Safety (ANSES), 105 route des Chappes, 06902 Sophia Antipolis cedex, France

**Keywords:** *Apis mellifera*, *Varroa destructor*, Deformed wing virus, Loads, Transmission, Offspring, Variant A, Variant B

## Abstract

**Background:**

*Varroa destructor* is the major ectoparasite of the western honey bee (*Apis mellifera*). Through both its parasitic life-cycle and its role as a vector of viral pathogens, it can cause major damage to honey bee colonies. The deformed wing virus (DWV) is the most common virus transmitted by this ectoparasite, and the mite is correlated to increased viral prevalence and viral loads in infested colonies. DWV variants A and B (DWV-A and DWV-B, respectively) are the two major DWV variants, and they differ both in their virulence and transmission dynamics.

**Methods:**

We studied the transmission of DWV between bees, parasitic mites and their offspring by quantifying DWV loads in bees and mites collected in in vitro and in situ environments. In vitro, we artificially transmitted DWV-A to mites and quantified both DWV-A and DWV-B in mites and bees. In situ, we measured the natural presence of DWV-B in bees, mites and mites’ offspring.

**Results:**

Bee and mite viral loads were correlated, and mites carrying both variants were associated with higher mortality of the infected host. Mite infestation increased the DWV-B loads and decreased the DWV-A loads in our laboratory conditions. In situ, viral quantification in the mite offspring showed that, after an initially non-infected egg stage, the DWV-B loads were more closely correlated with the foundress (mother) mites than with the bee hosts.

**Conclusions:**

The association between mites and DWV-B was highlighted in this study. The parasitic history of a mite directly impacts its DWV infection potential during the rest of its life-cycle (in terms of variant and viral loads). Regarding the mite’s progeny, we hypothesize that the route of contamination is likely through the feeding site rather than by vertical transmission, although further studies are needed to confirm this hypothesis.

**Graphical Abstract:**

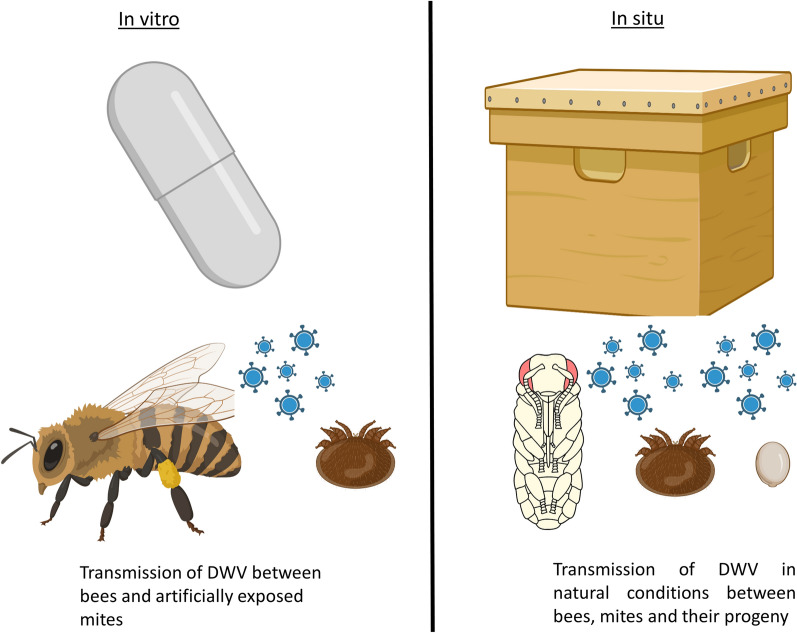

**Supplementary Information:**

The online version contains supplementary material available at 10.1186/s13071-022-05463-9.

## Background

In any host–pathogen or host–parasite relationship, the mode of transmission determines the dynamics and virulence of the pathogen [1–3]. Vector-borne or, more broadly speaking, horizontally transmitted pathogens are generally thought to be more harmful to their host than their vertically transmitted counterparts because they do not depend directly on host fitness to reproduce and infect new individuals [4–8]. Regarding vector-borne diseases, the increase in virulence may be due to the direct injection of the pathogen by the vector, causing systemic infection of the host. This direct injection could allow the pathogen to bypass host defense barriers and facilitate access to replication sites, resulting in higher loads and/or in the emergence of detrimental symptoms [4, 6, 9].

Many parasitic arthropods, such as mosquitoes, flies, fleas and ticks, are well-known vectors of viral, bacterial and unicellular pathogens [10–13]. Among those vectors, *Acari* taxa are well represented, with more than 800 species of parasitic ticks and several entire mite genera embracing the parasitic lifestyle [14]. One mite species in particular, *Varroa destructor* (Anderson and Trueman), has drawn the attention of scientists owing to its tremendous effects on a key pollinator: the western honey bee (*Apis mellifera*) [15]. *Varroa destructor* is an ectoparasite of both its original host *Apis cerana* and the sister species *A. mellifera* and is well-known to act as a vector of several viruses [16–19].

Among the 70 viruses that have been identified in honey bees, only a few have been associated to symptoms [17, 20–22]. At least three of these viruses with known symptoms, namely the acute bee paralysis virus, the deformed wing virus (DWV) and the slow bee paralysis virus, are closely associated with the presence of *V. destructor* [23–25]. DWV is the most prevalent virus in apiaries worldwide and is implicated in colony losses due to its deleterious symptoms [23, 26]. Transmission of DWV between bees has been shown to occur in a variety of different ways (from egg-laying by infected queens to food consumption in workers), but *V. destructor* is clearly a compounding factor [17, 27]. Evidence shows that because the mite injects viral particles directly into the hemolymph of its host when feeding, *V. destructor* parasitism leads to an increase in DWV viral loads and an initial decrease in DWV diversity at the colony level [28–30]. The immunosuppressive effect of the parasite, probably due to the consumption of fat bodies, is also a factor facilitating DWV infection in bees [31]. *Varroa destructor* could in fact be related to the selection of DWV variants that are vectored efficiently, which could make the *Varroa*-virus association only more detrimental. However, the initial bottleneck selecting one or a small number of variants may be followed by recombination events and a diversification phase [32].

Four master DWV variants have been described to this date, namely DWV variants A, B, C and D [33–35]. Many recombinants between these variants have also been identified, which increases the diversity of this viral quasispecies [33, 36, 37]. Among the major variants, DWV variant B (DWV-B) is more prevalent in Europe, whereas DWV variant A (DWV-A) is widespread in North America [32, 38–41]. On several occasions, DWV-B has been shown to outcompete DWV-A in laboratory experiments and field surveys [41–45], although the results seem to depend on the methodology followed in the study [46]. The rapid spread of DWV-B in Hawaii following the recent mite invasion also suggests a strong selective advantage of this variant over DWV-A when the parasite is present [28, 45]. After years of debate, it now appears that DWV-B is infectious to mites, whereas DWV-A is transmitted by mites in a non-propagative manner [47–49]. The emergence of symptomatic bees in a colony is probably more closely related to bee viral loads and the replication site within the bee body than to the variant [34]. However, the severity of symptoms and the lethality of the infection to a colony is often thought to be variant-related, even though diverse conclusions have been drawn in studies [36, 40, 42, 44, 50–53].

Many studies have focused on the dynamics of DWV replication in bees or between the parasitic vector and the bee. However, to our knowledge, none have specifically focused on the transmission between the mite foundress and their offspring or on the effects of DWV variants on mite survival and reproduction. The transmission dynamics are particularly relevant now that there is evidence that some variants can replicate and infect *V. destructor* [47, 48, 54]. The objective of the present study was to investigate these aspects through both in vitro and in situ studies. In laboratory conditions, we set out to measure the effect of co-infection with two DWV variants (genotypes DWV-A and DWV-B) during the *V. destructor* reproductive phase. In natural conditions, the purpose was to investigate the transmission of DWV between *V. destructor* foundresses and their offspring. More precisely, this study aimed to link the DWV loads of immature mites, either with their mother or with the bee they parasitize, and to identify the infectious stages in the mite life-cycle.

## Methods

### In vitro study of the transmission and virulence of DWV-A in association with DWV-B during the reproductive phase of* V. destructor*

#### Biological material

In November 2018, one *A. mellifera* (Buckfast) colony from the ANSES laboratory (French Agency for Food, Environment and Occupational Health & Safety, Sophia Antipolis, France) was treated for 1 month with the synthetic acaricide amitraz (Apivar; Véto-pharma, Palaiseau, France), followed by a final oxalic acid treatment in December 2018. After the second treatment, the colony was transferred to an indoor apiary. The indoor conditions allowed for shortened wintering and early resumption of queen egg-laying, and allowed for a negligible *V. destructor* infestation [50]. No natural mite fall was observed in the colony during the whole period of bee collection until the end of March 2019. Moreover, at the end of the experiments, honey bees emerging from the indoor colony were tested to quantify their natural viral loads. At this time, a pre-existing DWV-B infection was detected in this theoretically healthy colony. The persistence of the DWV-B infection in the absence of *V. destructor* infestation could be related to oral or vertical transmission within the colony [55–57].

DWV-A and DWV-B viral suspensions were obtained from naturally infected honey bees collected from Italy and Croatia, respectively. The inocula were adjusted in phosphate buffer (PB) as previously reported [50]. The viral loads in the undiluted suspensions were quantified using real-time quantitative PCR (RT-qPCR) and sequenced (Additional file 1: Text S1; Additional file 1: Table S1).

An *A. mellifera* colony naturally infested with mites (from an outdoor apiary) was used to provide the *V. destructor* foundresses. The foundresses were collected on drone pupae from sealed brood frames.

#### Artificial transmission of DWV to* V. destructor*

**Fig. 1:**
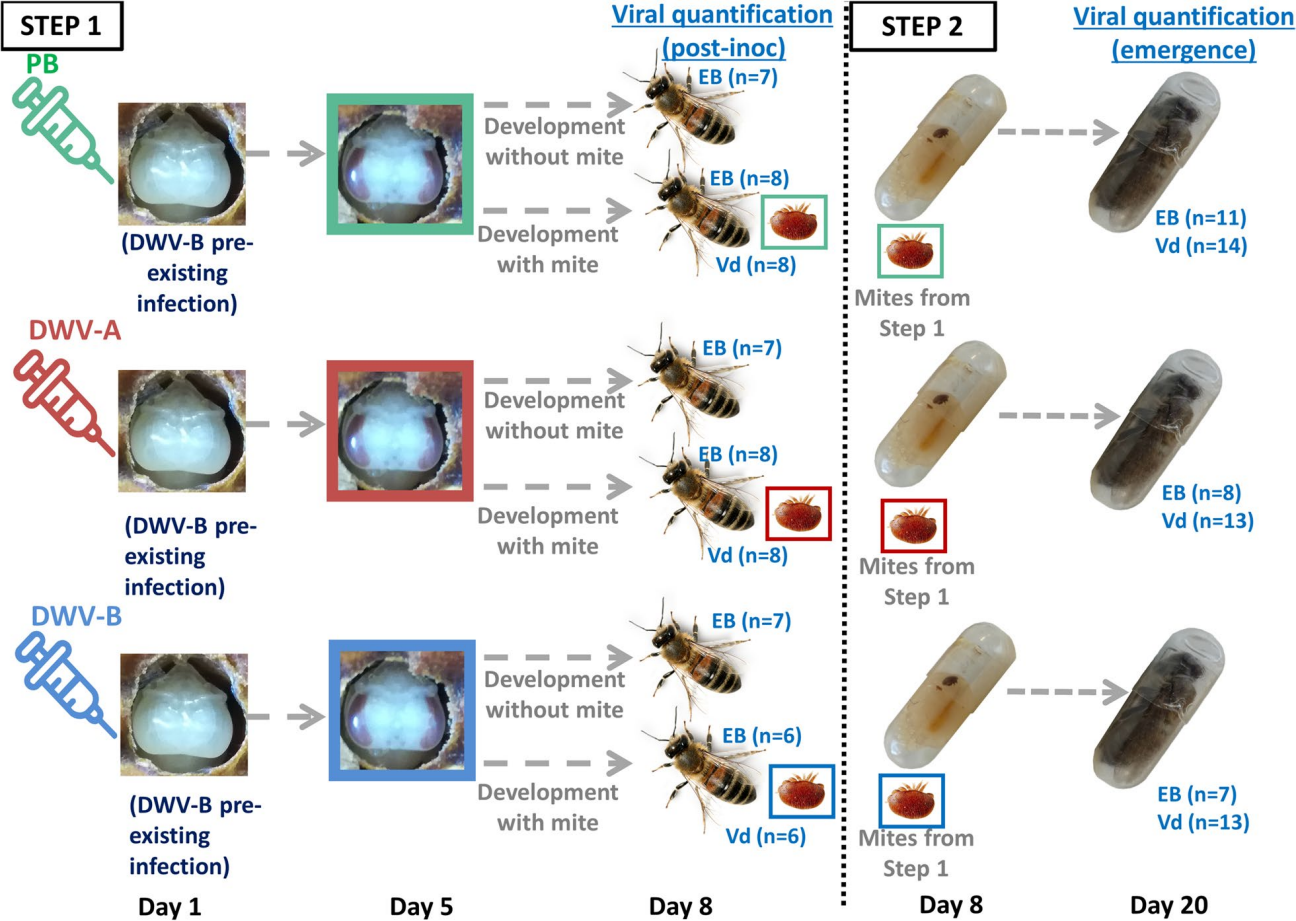
Protocol developed for studying the transmission of DWV between *Varroa destructor* (Vd) females and honey bees (HB) in in vitro conditions. During step 1, white-eyed pupae were inoculated with 5 × 10^2^ copies of DWV variant A or B (DWV-A or DWV-B), or injected with control phosphate buffer (PB). After 4 days, a total of 62 mites sampled from the infested colony were transferred to the treated brown-eyed pupae (22 mites on 22 PB-injected pupae, 21 mites on 21 DWV-A-inoculated pupae and 19 mites on 19 DWV-B-inoculated pupae). Nine additional mites were also sampled to check their initial DWV status, and 21 bee pupae were left without mites (7 per DWV condition). The mites and pupae were kept in an incubator (34.5 °C, 70% relative humidity) for 4 additional days until the bee reached the imaginal stage. A sample of 22 mites and bees was frozen for later molecular analysis (8 in the PB control, 8 in the DWV-A treatment and 6 in the DWV-B condition) along with the 21 *Varroa*-free bees. The remaining 40 mites (14 in the PB control and 13 in each of the two test conditions) were used in a second step and transferred to untreated fifth instar larvae from the healthy colony in gelatin capsules. The capsules were then kept in an incubator until emergence. These bees (when alive) and mites were then sampled and stored at − 80 °C until molecular analyses were performed. The analyses later revealed a pre-existing DWV-B infection interfering with our treatments. DWV, Deformed wing virus; EB, emerging bees

As a first step, we artificially injected white-eyed honey bee pupae from the uninfested colony with either PB (control) or purified viral solution of DWV-A or DWV-B (Fig. 1). To do so, 100 white-eyed pupae were first collected on two brood frames from the indoor colony and kept for 2 h in an incubator to check for any possible lesion caused by sampling.

The pupae were then placed individually on sterile filter paper in the wells of 24-well plates and assigned to one of the following three treatments: pupae injected with 2 µl of PB solution (control treatment, *n* = 32), pupae inoculated with 500 copies of DWV-A suspended in 2 µl of PB solution (*n* = 32) and pupae inoculated with 500 copies of DWV-B suspended in 2 µl of PB solution (*n* = 32). The pupae were injected in the ventrolateral region of the abdomen using a Hamilton syringe according to Mockel et al. [55]. Before and after use, the syringes were washed 3 times with PB, 3 times with 70% EtOH and again 3 times with distilled water.

The treated honey bee pupae were kept in an incubator (34 °C, 70% relative humidity [RH]) and checked 24 h after injection. Those pupae showing signs of sustained injury due to the injection were discarded. The healthy injected pupae were kept in the incubator until viral replication reached a plateau and there were > 10^6^ copies/pupa [44, 53]. On the 5th day after injection, a total of 62 brown-eyed pupae from the three groups were transferred into 0.33-ml gelatin capsules (LGA, La Seyne-sur-Mer, France) [58], and each of the capsules was artificially parasitized with one mite foundress (Fig. 1, step 1). The rest of the treated pupae remained uninfested and were kept in the 24-well plates. A sample of nine mites was also collected and stored in the freezer to determine the initial viral status of *V. destructor*. The parasitized pupae in capsules and the *Varroa*-free pupae in plates were kept for 4 additional days in an incubator until emergence of adult bees (Fig. 1). On the day of emergence, a total of 22 infected bees and their corresponding mites were collected and stored at − 80 °C until quantification was performed to analyze virus replication (PB control *n* = 8, DWV-A treatment *n* = 8, DWV-B treatment *n* = 6; Fig. 1). To check the success of our artificial inoculation, 21 *Varroa*-free pupae (7 per condition) were also frozen and kept at − 80 °C until further molecular analyses. The remaining 40 foundress mites that were not collected for virus quantification were used in the subsequent in vitro rearing bioassay (14 mites from the control PB group, 13 from the DWV-A group and 13 from the DWV-B group; Fig. 1, step 2).

#### In vitro rearing of artificially infected *V. destructor*

Two brood frames from the uninfested indoor colony were taken to the laboratory and fifth instar larvae from unsealed cells were transferred into 0.33-ml gelatin capsules as described in [59, 60]. The 40 V*. destructor* females retrieved at the end of the artificial infection experiment were introduced into the capsules containing healthy bee larvae. The capsules were placed in an incubator (34 °C, 70% RH) until emergence around the 12th day. The parasites and the hosts were collected and kept at − 80 °C until further analyses (see Fig. 1, step 2). Unfortunately, virus loads could not be quantified in several of the dead bee pupae (*n* = 14) due to damage or failed development caused by the in vitro parasitization. Because parasite reproduction was low (see Results section), the number of offspring was not sufficient to reach a relevant number of replicates for analysis. The relation between the viral loads in foundresses and their offspring was therefore tested in an in situ experiment.

### In situ study of the transmission between bees, mites and their offspring during the reproductive phase of* V. destructor*

#### Biological material

Three naturally infested honey bee colonies of Buckfast-Carniolan origin were used in the in situ study. The colonies were kept on the university campus (Albi, France) and occasionally fed with sucrose syrup. Bees and mites from these colonies were sampled between August and September 2019.

#### Sampling of mites and bees

Four developmental stages of bees were considered in this study, which aimed to investigate the dynamics of viral abundance in the host-parasite-progeny triad throughout *A. mellifera* development. These four honey bee pupal stages and the type of *V. destructor* offspring sampled are described in Table 1.

**Table 1:** Developmental stages of the bee pupae and corresponding stages of the parasite (foundress + juvenile offspring) collected to quantify the viral loads

Sampled honey bee stage	Day post-capping	*Varroa destructor* stages sampled	Sample size (*n*)
White-eyed pupa	D + 4	Foundress + egg	14
Pink-eyed pupa	D + 6	Foundress + protonymph	16
Pupa (light pigmentation of the cuticle)	D + 9	Foundress + deutonymph	14
Pupa (dark pigmentation of the cuticle)	D + 11	Foundress + adult daughter	15

For sampling, sealed brood frames from the three honey bee colonies were taken to the laboratory. The capped cells were opened with tweezers and the bee pupae were carefully removed. Only those individuals parasitized by one successfully reproductive *V. destructor* foundress and its offspring were collected into 1.5-ml microcentrifuge tubes. The corresponding foundress mite and the egg or oldest female offspring were also sampled in separate 1.5-ml microcentrifuge tubes and were given matching sample codes. A total of 59 bee samples with their corresponding mites were collected, immediately frozen and stored at − 80 °C until further molecular analyses (Table 1).

### Molecular analyses

#### RNA purifications and reverse transcription

Total RNA of *V. destructor* samples was purified from 50 µl of homogenates of adult and immature stages using the NucleoSpin RNA-Mini kit (Macherey Nagel, Düren, Germany), following the manufacturer’s instructions. The 260:280 absorbance ratios (A260:A280) were measured using a BioTek Instruments analyzer (Winooski, VT, USA) analyzer. For the bee samples, total RNA was purified according to the manufacturer’s instructions from 140 µl of clarified pupa head homogenates (1 bee head per 500 µl PB) using the QIAamp Viral RNA Mini Kit (Qiagen, Hilden, Germany). Viral particles were quantified in bee-pupae heads as previous studies highlighted that this is a clinical sign of overt DWV infection [36, 55, 61]. The extracted RNA was eluted from a spin column in 60 µl of DEPC-treated water or elution buffer (Qiagen). The RNA recovery rate from bee-head sample was not estimated because carrier RNA was used to enhance RNA purification efficiency. Next, 11 µl of pure undiluted RNA extract (about 159 ng of mite RNA) was transcribed into first-strand complementary DNA (cDNA) using the Superscript IV First-Strand Synthesis System (Thermo Fisher Scientific, Waltham, MA, USA). The final volume of 20 µl of cDNA was kept at − 20 °C until the final quantification step was performed.

#### Quantification by RT-qPCR

The DWV-A and DWV-B quantifications (genotype specificity based on VP3 coding sequence; Additional file 1: Table S2) were performed in the ANSES laboratory (Sophia Antipolis, France), following the method described in Schurr et al. [62]. The amplification reaction was performed in a MicroAmp optical 96-well reaction plate in a total reaction volume of 25 μl containing 1× TaqMan® Universal PCR Master Mix with uracil-N-glycosylase (UNG) (2X; Applied Biosystems, Thermo Fisher Scientific), 320–1200 nM of each forward and reverse primer, 100–400 nM of the probe, 1× Exo IPC Mic VIC (Applied Biosystems, Thermo Fisher Scientific), 1× Exo IPC DNA (Internal Positive Control; Applied Biosystems, Thermo Fisher Scientific) and 5 μl of cDNA template. Threshold cycles (Ct) from our samples were compared with standard curves to establish a linear relation between Ct and plasmid loads in the range of 1.0 × 10^2^ to 1.0 × 10^8^ copies/5 μl. In the case of the occasional contamination of negative controls with low numbers of residual DWV particles, a strict correction based on the highest control load detected was applied. Final results were expressed in log_10_ equivalent viral genome copies (log_10_ genome copies/bee head and log_10_ genome copies/mite). For each sample, the dilution factor was taken into account based on the proportions of volumes used at each step of the method (sample preparation, RNA extraction, RT-qPCR (Additional file 1: Table S3). The results were thus quantified as viral copy numbers per bee or per mite. For the quantification of DWV-A and DWV-B in mites, the reproducibility of the viral loads was calculated taking into account the ratio of RNA recovery (Additional file 1: Figure S1). The overall variability of RNA amounts at the end of the extraction (explained for the most part by the developmental stage of the mite) had a low effect on the quantification reproducibility. For the DWV-A and DWV-B quantifications in the bee head, the precision and the reliability of the viral loads were assessed through the construction of accuracy profiles (Additional file 1: Figure S2), according to de Miranda et al. [17]. In both mites and bees, the tolerance limits of the methods were within the range of ± 1 log_10_ copies per mite or per bee head, respectively.

#### Statistical analyses

Results were analyzed using R.4.0.4® (Core Team 2021; R Foundation for Statistical Computing, Vienna, Austria) and graphs were generated using the ggplot2 package [63].

The results on viral loads of bees and mites from the in vitro study were first explored using a principal component analysis (PCA) on the whole dataset (including both post-inoculation and emergence data (see Fig. 1). DWV loads of parasitized and mite-free bees 8 days after their injection (post-inoculation) were compared using Mann–Whitney–Wilcoxon tests. Spearman correlation tests were run on viral loads to investigate the links between mite and bee viral loads, for both DWV-A and DWV-B. Kruskal tests were also performed to analyze the viral loads of mites in relation to their treatment group. When significance was detected, pairwise comparisons were further performed using Mann–Whitney-Wilcoxon tests and the* P* values were adjusted based on the Bonferroni correction. For DWV-A, artificial infection was successful, and the effect of sampling time (before the start of the experiment (J0), post-inoculation and after emergence) on the loads carried by the mite was evaluated using the same ranking analyses.

Binary data resulting from the final in vitro rearing, including bee mortality, mite mortality and mite oviposition, were all analyzed using generalized linear models (GLMs). Mite mortality and oviposition were analyzed using mite DWV-A and DWV-B loads along with the survival of bees as explanatory variables. Bee mortality was also investigated. Although interesting, the viral loads of bees could not be included as an explanatory variable in this latter analysis due to the bias caused by some of the dead bee pupae whose viral loads could not be quantified because they were too damaged (not fully developed pupa head). Therefore, the DWV-A and DWV-B loads in mites and a qualitative variable categorizing the mites as being vector of one or two DWV variants were the three dependent variables tested in the statistical model.

Regarding the in situ data, analysis of the mite offspring viral loads was conducted using a linear model because the distribution of the residuals met all the assumptions associated with this model. In this case, the bee viral loads, mite/bee developmental stage (Table 1) and the identity of the colony sampled were all included as dependent variables. The bee and foundress viral loads were analyzed using Spearman correlation tests.

## Results

### In vitro study of the transmission and virulence of DWV-A in association with DWV-B during the reproductive phase of* V. destructor*

#### Viral status of bees 8 days after DWV inoculation to bee pupae

**Fig. 2:**
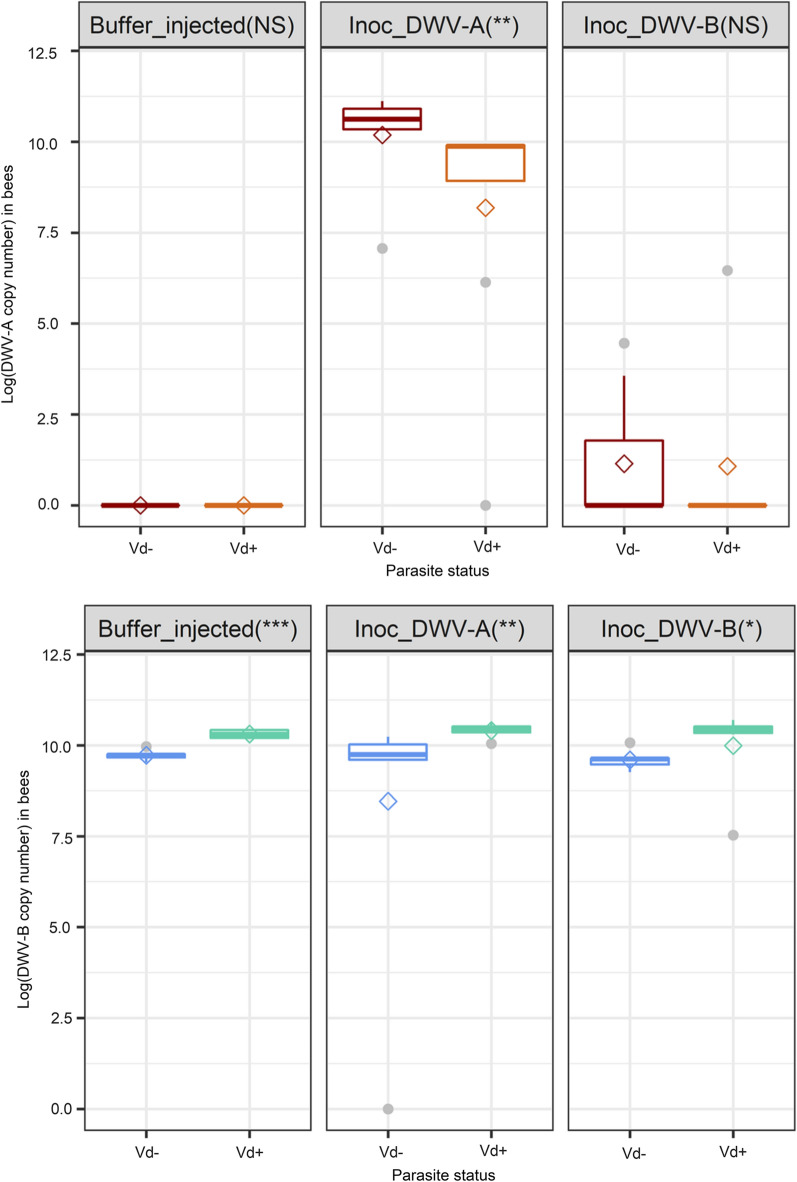
Boxplot showing the log-transformed virus copy numbers of DWV-A (upper panel) and DWV-B (lower panel) in parasitized (Vd+) and mite-free (Vd− ) bees 8 days post-inoculation. The pre-existing DWV-B infection of pupae resulted in high DWV-B copy number in all three groups, as clearly visible in the left panel. The asterisks in the header cells indicate the level of significance: *P ≤ 0.05; **P ≤ 0.01; ***P ≤ 0.001; NS, not significant. Diamonds indicate the mean value of the log_10_-transformed viral copy numbers

The analyses showed that the mites collected in March 2019 before the start of the experiments were naturally infected with DWV-B (mean load: 4.0 ± 1.4 log_10_ copies/mite), whereas DWV-A was not detected (Additional file 1: Table S1; Additional file 1: Figure S3). The mite-free honey bee pupae from the control PB treatment all displayed high DWV-B loads whereas DWV-A was not detected (Additional file 1: Table S1; Additional file 1: Figure 2). The natural presence of DWV-B at high loads in the colony was confirmed by quantification of viral loads in bees collected at the end of the experiment (DWV-B load: 10.82 log_10_ copies/bee). Subsequently, the DWV-B loads remained unchanged in bee pupae inoculated with DWV-A (collected in March 2019) or DWV-B (Fig. 2). On the contrary, an increase in the DWV-A load was only observed in DWV-A-inoculated bees, not in DWV-B-inoculated bees or control bees (Fig. 2). Despite the presence of low residual loads of sacbrood virus (SBV) in the DWV inocula, no infection of the inoculated pupae was observed in our study. The SBV was scarcely detected in the inoculated bees 8 days after injection (Additional file 1: Table S1).

Interestingly, the comparison between mite-free and parasitized emerging bees 8 days post-inoculation highlighted that the bee DWV loads depended significantly on the presence of *V. destructor*. In bees inoculated with DWV-A, mite presence significantly decreased the DWV-A viral loads (Wilcoxon test, *W* = 50, *P* ≤ 0.01) and in all groups, mite presence increased the DWV-B viral loads (Wilcoxon test, buffer-injected bees* W* = 0,* P* ≤ 0.001; DWV-A-inoculated bees *W* = 2, *P* ≤ 0.01; DWV-B-inoculated bees *W* = 7, *P* ≤ 0.001; Fig. 2).

#### Evolution of viral loads in mites during the reproductive phase of *V. destructor* and transmission to untreated bees

**Fig. 3:**
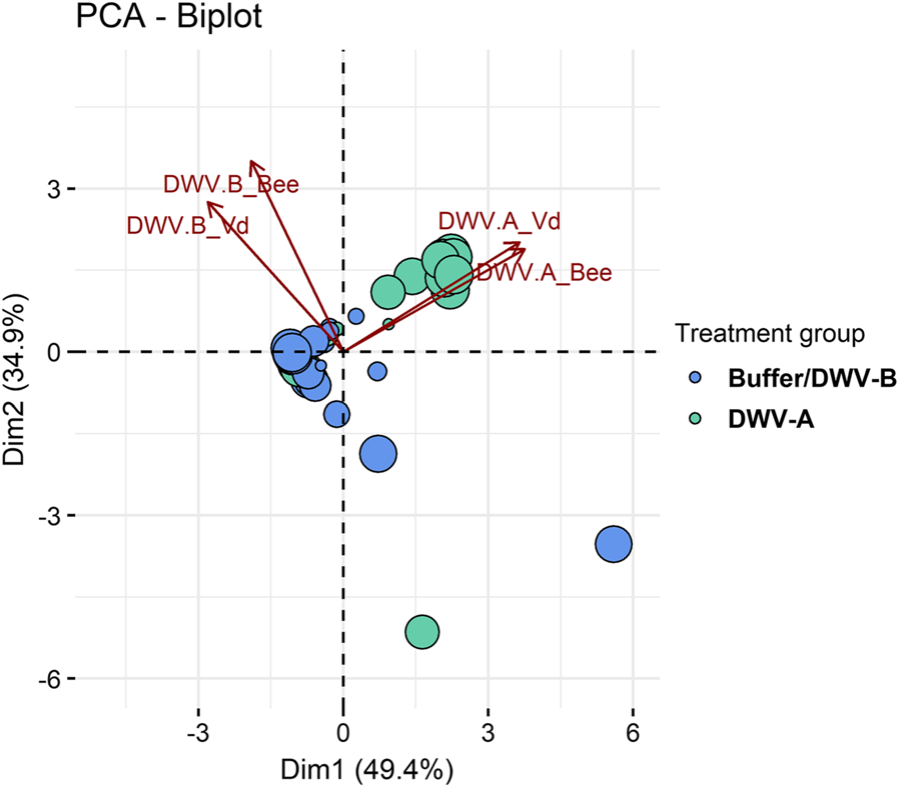
Principal component analysis biplot of DWV variant quantifications in mites (Vd) and in bees. The bees and mites from the three different treatments (inoculation with DWV-A, with DWV-B or with PB solution) were analyzed at two time points: right after the inoculation period and at the end of the in vitro rearing period (see Fig. 1). The pre-existing DWV-B infection of pupae resulted in high DWV-B copy number in all three groups so the DWV-B-inoculated and buffer-injected groups were pooled together on this graph since they had the same viral profiles

The DWV loads of pupae were always significantly correlated with the mites that parasitized them, regardless of the DWV variant (Fig. 3; DWV-B: Spearman’s *r* = 0.31, *P* ≤ 0.05, *n* = 62; DWV-A: Spearman’s *r* = 0.66, *P* ≤ 0.001, *n* = 62). However, the correlation was found to be less strong for DWV-B as high loads were always detected regardless of the treatment. The DWV-B loads in mites parasitizing buffer-injected, DWV-A- or DWV-B-inoculated pupae matched the profile of their host and were not significantly different between the three groups (Fig. 2; Additional file 1: Figure S3; Kruskal test, *χ*^2^ = 2.57,* df* = 2, *P* = 0.28). Nevertheless, DWV-B loads in mites from the three treatment groups were significantly higher than the initial mite viral loads before their stay on artificially inoculated pupae (Kruskal test, *χ*^2^ = 14.70,* df* = 3, *P* ≤ 0.01; J0 mites vs DWV-B-inoculated mites: Wilcoxon, *W* = 20, adjusted-*P* ≤ 0.01; J0 mites vs DWV-A-inoculated mites: Wilcoxon, *W* = 23, adjusted-*P* ≤ 0.01; J0 mites vs PB-buffer-injected mites: Wilcoxon, *W* = 29, adjusted-*P* ≤ 0.05).

On the contrary, the DWV-A loads in mites matched the profiles of their host and depended significantly on the inoculation treatment. Consequently, higher loads were obtained in *V. destructor* females when they had parasitized DWV-A inoculated bee pupae (Additional file 1: Figure S3; Kruskal test, *χ*^2^ = 26.80,* df* = 2, *P* ≤ 0.001; DWV-A vs DWV-B inoculated: Wilcoxon, *W* = 342, adjusted-*P* ≤ 0.001; PB vs DWV-A inoculated: Wilcoxon, *W* = 72, adjusted-*P* ≤ 0.001; DWV-B vs. buffer-injected: Wilcoxon, *W* = 215, *P* = 0.83).

These results are highlighted in the PCA biplot (Fig. 3). The DWV-A viral loads are mostly represented on axis 1 (cos2_DWV-A bee = 0.71, cos2_DWV-A Vd = 0.68), whereas the DWV-B loads are represented on axis 2 (cos2_bee = 0.62, cos2_Vd = 0.38). As shown by the placement of viral load variables, the DWV-A and DWV-B copy numbers are correlated between mites and bees. The vast majority of individuals from the DWV-A-inoculated group, whether mites or bees, all showed high loads of DWV-A, which appears in the positioning of individuals along the DWV-A variables (Fig. 3). The individuals from the DWV-B- or PB-injected groups were in fact more characterized by the absence of DWV-A rather than by the presence of DWV-B, which explains their more central position on the Dim 1 axis (Fig. 3).

**Fig. 4:**
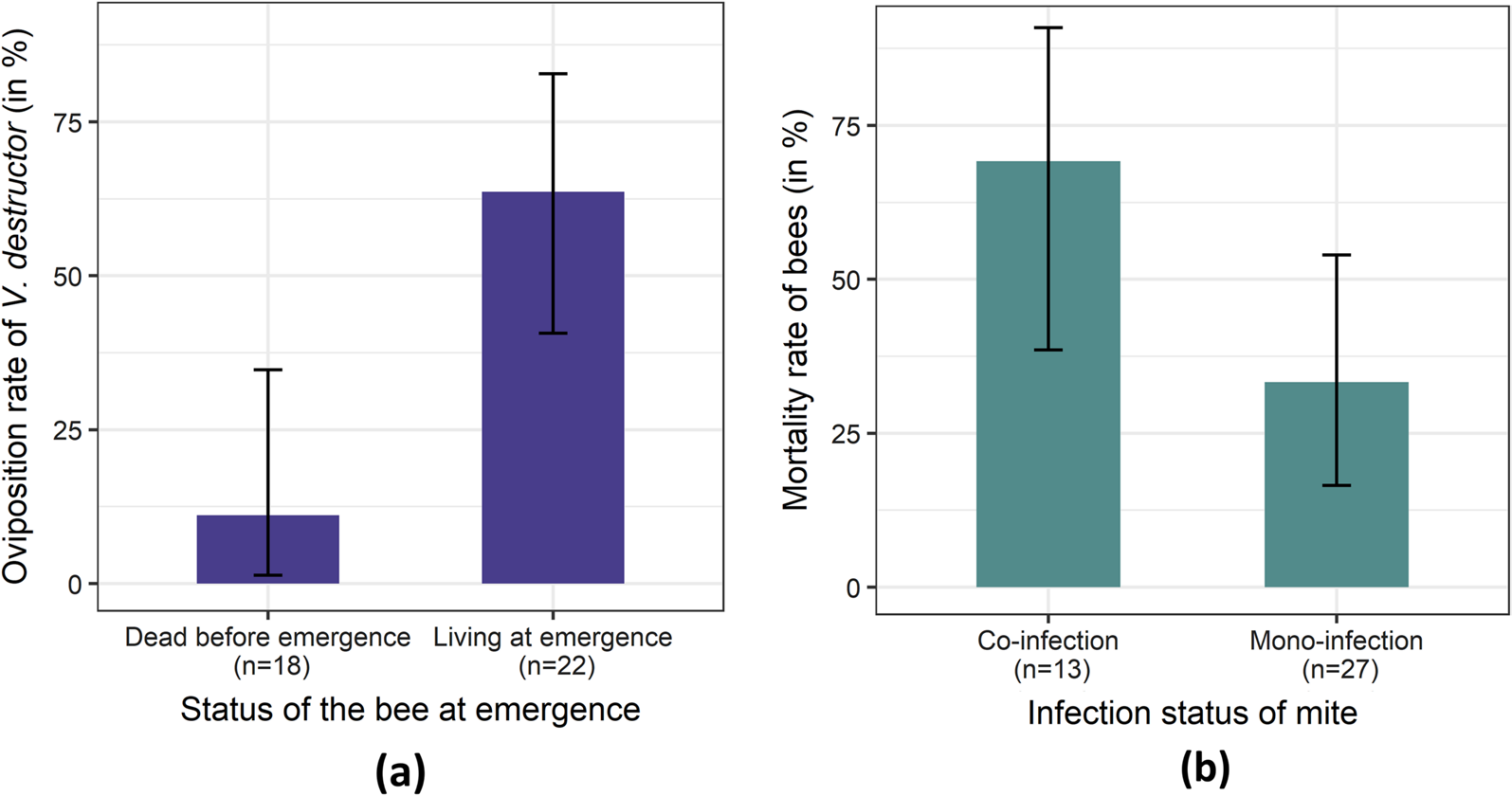
**a** Oviposition rate ± 95% confidence interval (95% CI) of mites according to bee survival on the day of emergence (*P* ≤ 0.001). **b** Mortality (± 95% CI) of bees parasitized by *V. destructor* foundresses vectoring either DWV-B (Mono-infection) or both DWV-A and DWV-B (Co-infection). *P* ≤ 0.05

During the in vitro rearing on uninjected bee larvae that followed the artificial transmission of DWV to mites (Fig. 1, step 2), mite mortality (27.5%; 95% confidence interval [CI] 14.60–43.89) and low reproduction (40%; 95% CI 24.87–56.67) did not vary significantly with treatment. More precisely, in our conditions, neither mortality (GLM mortality: deviance = 1.26, df = 2, *p* = 0.53) nor oviposition of *V. destructor* (GLM oviposition: deviance = 1.16, df = 2, *p* = 0.56) was significantly affected by the DWV-A or DWV-B loads. Bee survival, on the other hand, significantly affected mite oviposition success (GLM oviposition: deviance = 12.44, ddf = 1, *p* ≤ 0.001) and egg-laying obviously occurred more frequently when the bee survived the entire mite reproductive phase (Fig. 4a). Although it would have been interesting, mite offspring viral loads could not be investigated in the in vitro rearing experiment due to excessively low reproductive success. Interestingly, when we categorized the mites as being either a vector of DWV-B (mono-infection) or of DWV-A and DWV-B (co-infection), the mortality rate of bees was significantly higher when mites carried both DWV variants (GLM: deviance = 4.63,* df* = 1, *P* ≤ 0.05). Even though viral loads were considered, only the co-infection effect was retained in the final model. The transmission of both DWV variants by *V. destructor* thus increases the lethality of the virus (Fig. 4b) and indirectly lowers mite reproduction.

### In situ study of the transmission between bees, mites and their offspring during the reproductive phase of *V. destructor*

The quantification of DWV did not reveal the presence of DWV-A in our collected samples from three *A. mellifera* colonies whereas high loads of DWV-B were frequently detected (Additional file 1: Figure S4).

**Fig. 5:**
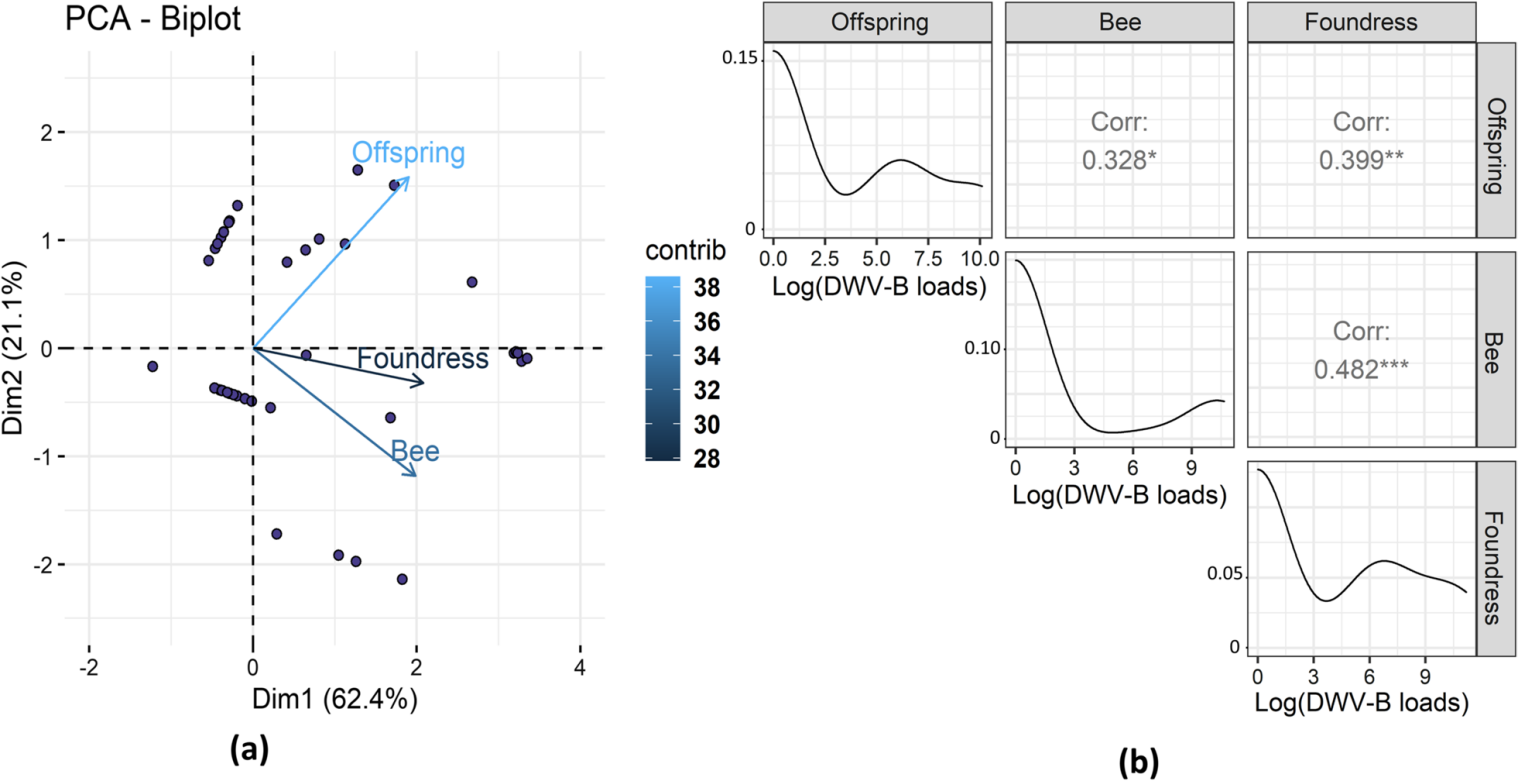
**a** Biplot graph from a PCA showing the positive correlation between the log_10_-transformed copy numbers of DWV-B measured in *V. destructor* foundresses, in their offspring and in bee pupae. Each dot combines the viral copy numbers quantified in the bee and the mites sampled in a single cell. The contribution of the variables to the first axis is represented by the color gradient and is around 35% for each of the variables. **b** Distribution of the log_10_-transformed DWV-B copy numbers in bees, mite foundresses and their offspring along with the Spearman correlation rates measured between variables. Asterisks indicate the level of significance: *P ≤ 0.05; **P ≤ 0.01; ***P ≤ 0.001. PCA, Principal component analysis

The distributions of viral loads were slightly different between the parasite and the host. In bees, there was a bimodal distribution with either null or high loads of viral particles measured, whereas intermediate loads often occurred in both juvenile and foundress mites (Additional file 1: Figure S4; Fig. 5b). It is also interesting to note that several bees (17/59) were DWV-B-free even though the *V. destructor* foundresses were infected with the virus. Despite these divergences, the DWV-B loads of the bees and foundresses were significantly correlated (Fig. 5; Spearman’s *r *= 0.48, *P* ≤ 0.001, *n* = 59). This correlation seems to be pupal-stage-dependent as the later dark pigmented stage is associated with an absence of correlation.

**Fig. 6:**
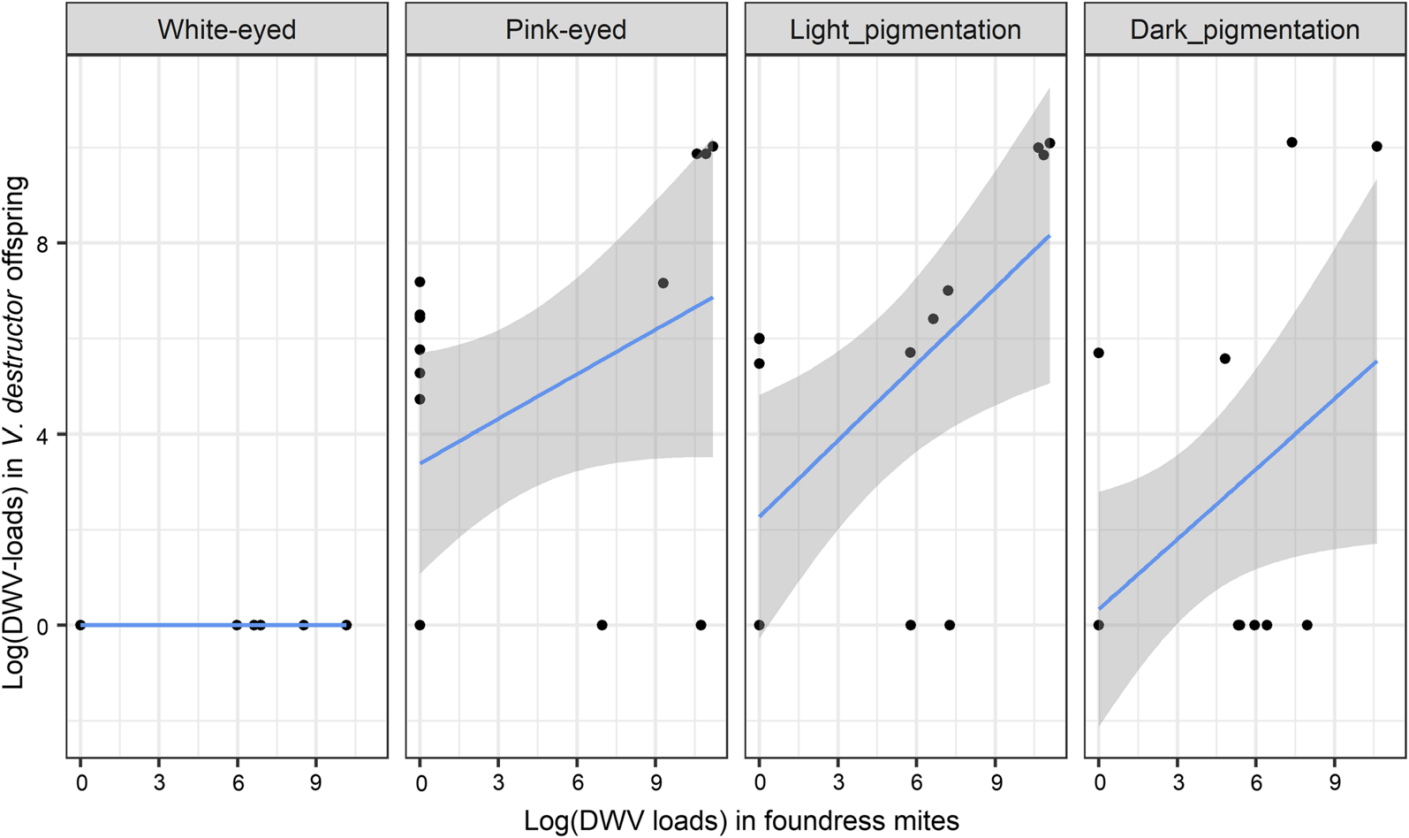
Relationship between DWV-B copy numbers of *V. destructor* in mother mites (foundress mites) and the viral copy numbers of their offspring, according to bee pupal developmental phase

The offspring viral loads were significantly correlated to the bee viral loads (Spearman’s *r* = 0.33, *P* ≤ 0.05, *n* = 59) and to a greater extent to the foundress viral loads (Spearman’s *r* = 0.40, *P* ≤ 0.05, *n* = 59; Fig. 5). This is shown in Fig. 5a where the three viral quantification variables are correlated on axis 1 (which summarizes 62% of the data variance). Despite the positive correlation between the loads in bees and those in mite offspring, the infection of the mite protonymphs, deutonymphs and daughters appeared to be more related to the DWV-B loads in their mother than to those in the bee pupae they parasitized (Figs. 5, 6). In the final model predicting the offspring infection loads, we thus retained the foundress viral load rather than the bee viral load. In addition to the foundress viral loads (linear model,* df* = 1, *F *= 10.75, *P* ≤ 0.01), the bee developmental phase (*df* = 3, *F* = 7.05, *P* ≤ 0.001) and colony identity (*df* = 2, *F* = 3.66, *P* ≤ 0.05) both significantly affected the viral loads found in *V. destructor* offspring. Interestingly, no DWV-B was detected in *V. destructor* eggs from the early white-eyed pupal stage, whereas it was detected in the subsequent developmental stages (protonymphs, deutonymphs and adults). In these cases, a positive correlation between the DWV-B loads in foundress and the immature stage of their offspring was always highlighted (Fig. 6).

## Discussion

### In vitro study of the transmission and virulence of DWV-A in association with DWV-B during the reproductive phase of* V. destructor*

#### Viral status of bees 8 days after DWV inoculation to bee pupae

Artificial transmission of DWV variants to *V. destructor* by parasitization of laboratory-inoculated honey bees can be impaired by the viral background of either the mite or the bee pupae, and has led to the use of genetically tagged DWV clones to study the virus [19, 32, 48, 49]. In our experiments, DWV-A was undetected or at low viral loads in pupae, and this DWV-A background did not interfere with interpretation of the results. Although DWV-B was detected before inoculation in pupae and mites, the viral dynamics of DWV-A (monitored using qPCR) could be investigated, along with the impact of co-infection on pupae and on mite reproduction. When DWV-A was injected into bee pupae, no short-term exclusion of either variant was observed in bees 8 days post-inoculation, and both variants were detected. In previous studies, without mites, competition between variants was shown to decrease DWV-B loads in co-injected bee pupae, but this decrease did not seem to lead to the exclusion of either variant and DWV-B still accumulated at higher levels than DWV-A [32, 44, 46]. In our case, the addition of *V. destructor* to inoculated bees also did not lead to competitive exclusion. However, parasitization resulted in higher DWV-B loads and lower DWV-A loads in parasitized bees compared with mite-free bees. The synergy between DWV-B and the mite led to higher viral loads, which might be associated with the replication of this variant in the mite’s body [47, 48]. The interactions between the mite, the non-infectious DWV-A and the infectious DWV-B could then result in a slight, but significant decrease in DWV-A loads via competition. Altogether, the dominance of DWV-B in European colonies does not appear to be related to direct short-term competitive exclusion of other DWV variants in bees. The replication of DWV-B in mites [48], food transmission between bees in the absence of *V. destructor* [64, 65] and more efficient replication than DWV-A [44] may be other factors explaining the dominance of DWV-B.

#### Evolution of viral loads in mites during the reproductive phase of *V. destructor* and transmission to untreated bees

Due to parasitization, the mites that fed on inoculated bees always acquired a matching viral profile. Because the honey bee pupae used in this study were naturally infected by DWV-B and not by DWV-A, parasites carried high loads of DWV-B regardless of treatment. On the contrary, the mites carried high loads of DWV-A only when the bee pupae they fed on had been inoculated with this variant. The fact that some mites initially carried intermediate loads of DWV-B had relatively little influence.

When compared with post-inoculation time, the DWV-A loads in mites decreased after the 12 days of the mite reproductive phase, although the difference was not significant (Additional file 1: Figure S3). Again, no clear competitive exclusion was thus observed in our study, as previously highlighted [44].

During this reproductive phase, mite reproduction was low compared to previous studies [59, 60]. For the most part, bee mortality associated with the virus explained the reduced reproduction of the mite. The parasite cycle is known to rely entirely on its host, and the mite cannot complete its reproductive phase properly without a healthy bee pupa [15, 25, 59, 66]. Although in our study, neither the DWV loads nor the presence of both DWV variants resulted in a significant decrease in *V. destructor* reproduction, the direct effect of DWV on mite reproduction cannot be excluded. DWV-A is not infectious for the parasite [49], whereas DWV-B clones were recently shown to replicate in mites [48, 67]. The consequences of viral replication in *V. destructor* females are not known yet, and further research is needed to fully understand the effect of DWV-B infection in mites.

Regarding the honey bee pupae, the DWV viral loads of mites did not have any significant influence on the bee mortality. Unfortunately, because some of the dead pupae were too damaged to be used in molecular analyses, we can only deduce any effects from the mite viral loads. Interestingly, however, the mites that carried both DWV variants appeared to cause bee mortality more frequently. This may indicate that, regardless of the loads, co-infection of parasitized bees with two DWV variants is deleterious to bees. Alternatively, the mere presence of DWV-A is sufficient to induce increased bee mortality, in which case this would be in agreement with several previous studies on the virulence of DWV-A [44, 51]. However, the bee mortality rate in the co-infection treatment was far higher than the rates reported in the literature [44, 50, 53]. Recently, Ray et al. [46] also observed high mortality after co-injection of DWV-A and DWV-B, with bee death generally occurring before emergence. The high mortality in our study may also be due to the presence of both DWV variants in addition to the parasite pressure of *V. destructor*. The presence of *V. destructor* is indeed associated with continuous injury to bees along with the likely injection of viral particles at the larval stage. Compared with pupae, the early developmental stages of bees may be more sensitive to stresses, as suggested by the high rates of deformity and mortality induced by DWV-fed larvae in the study of Gusachenlo et al. [48]. Despite the residual loads of SBV in the inocula, this virus was not frequently detected and even when it was, the viral loads remained low (Additional file 1: Table S1). These results seem to exclude that the deaths were due to the replication of SBV. Therefore, the high bee mortality throughout the three treatment groups was most likely due to the DWV-carrying parasites. Additional studies are needed to further assess the correlations and observations from the cohort sampled herein. In any case, this artificially induced high mortality is in fact counterproductive for the mite, because it does not allow the female to complete its reproductive phase [59, 60]. More generally, the viral history of a mite, i.e. its past exposure to infected bees, is an important factor to consider as it can impact the present mortality or reproduction of the parasite. In fact, each mite has its own infection potential based on the quantity and type of viral variants it carries, which in turn depends on the bees it recently parasitized. This represents a significant source of variability impacting the outcome of *V. destructor* field or laboratory studies [68].

### In situ study of the transmission between bees, mites and their offspring during the reproductive phase of *V. destructor*

Only DWV-B was detected in the three colonies used in this study. This result is in line with findings from previous studies showing that, in Europe, this variant has become the most prevalent variant in both feral and managed colonies [33, 38, 62]. As expected, the viral loads of bees, foundress mites and their offspring were all correlated. This is also in agreement with previous research focusing on viral replication in the bee-parasite pair [69]. However, in several cases, bee pupae parasitized by DWV-B-infected mites were surprisingly free of the virus, or at least infected at undetectable levels. In contrast, in the study of Wu et al. [69], viral particles were always detected in the bees parasitized by infected mites, even at low levels. Given that our RT-qPCR method is highly sensitive [62], the presence of undetectable loads of viruses seems unlikely, even though it cannot be ruled out. The absence of the virus may otherwise be due to immune mechanisms, such as RNA interference, allowing the bee to remain healthy despite being injected with viral particles coming from the mite [70–72]. This immune barrier would keep the infection at a covert stage, which could make it undetectable when quantifying from bee heads [36, 55, 61]. Furthermore, a bimodal distribution was observed in bees, with individuals showing either no or high loads of viruses, sometimes regardless of the mite loads, as previously described [69, 73]. This bimodal distribution could be explained by immune suppression beyond a certain threshold, as suggested by Di Prisco et al. [73], in which antiviral immunity is unable to control the replication of DWV, leading to high viral loads. These hypotheses were not directly tested in the present study and need further research.

The viral loads of the *V. destructor* offspring were more related to the mite foundress than to the bee pupal loads. Despite the initial mite egg stage being virus-free in our study, the correlation of the viral loads was strong between mites and their offspring. These results show that mite-associated viruses can occur early in the parasite’s life-cycle, as early as the protonymphal stage. The presence of viral particles at the nymphal stages of *V. destructor* has already been observed in the case of Kashmir bee virus [74]. In the present study, it appears that the offspring become infected when they are able to move and feed—and apparently not at the egg stage. Infection restricted to later nymphal stages would minimize the role of direct vertical transmission through foundress oviposition. Later, during the reproductive phase, the feeding site is maintained by the foundress to allow its immature offspring to feed. The shared contaminated feeding site could thus represent the most likely transmission route during the development of the mite [75]. This also means that the viral history applies to new female mites that can represent a viral infection potential as soon as they emerge from the newborn bee cell.

## Conclusions

Taken together, this *Varroa*-centered study on DWV variants shed light on viral transmission in mites. It would appear that mites become infectious as early as the juvenile stage and transmit the virus to bees. Bees seem able to control these viral loads injected by *V. destructor* and could remain asymptomatic even when parasitized by a DWV-infectious mite. We also confirmed that DWV-B was the naturally present variant in our colonies and demonstrated that mites carrying both DWV variants (DWV-A and -B) were more harmful to the bee. The viral infection potential of mites depends on their recent history. This infection potential has to be carefully considered as it is a source of variability that can impact the outcomes of studies. Further research is required to assess the impact of the infectious DWV-B on mites. A complementary sequencing approach would also provide further information on the DWV diversity present in this study.

## Supplementary Information

**Additional file 1: Text S1.** Genome sequence of both DWV-A and DWV-B inocula used for the in vitro experiments. The NEBNext rRNA depletion kit (New England Biolabs; Ipwich, MA, USA; E6310) and NEBNext Ultra II directional RNA kit (New England Biolabs; E7760) were used to prepare the sequencing libraries. Libraries were pooled and sequenced with Nextseq 500 (Illumina Inc., San Diego, CA, USA) using the 150-cycle high-output kit at the sequencing core facility of the Brain & Spine Institute, Paris, France. Average coverage depth was used to calculate the number of read to sample in order to get an average coverage depth of 80×. Subsample reads were de novo assembled using Spades (V3.10.0; Bankevich et al. [77]) into contigs and aligned to the reference genome to generate a consensus sequence. We are grateful to Fabrice Sircoulomb (ANSES Sophia Antipolis), Pierrick Lucas and Yannick Blanchard (ANSES Ploufragan) for performing the sequence analysis. **Table S1.** Viral copy number quantified by RT-qPCR in inoculated emerging honey bees, viral inocula (calculated viral copy numbers according to the dilutions injected) and foundress mites used in the in vitro study.** Table S2.** Primers, probe and recombinant plasmids used for RT-qPCR quantification according to Schurr et al. [63].** Table S3.** Factors used to convert the viral copy number per PCR into viral copy number per bee head or per mite. **Figure S1.** Distributions of *V. destructor* RNA amounts recovered after extraction, in relation to the stage of the mite considered. These variations in the total RNA amount were not taken into account because they had a limited impact on the general reproducibility of viral copy numbers quantified by RT-qPCR. Based on the ratios of RNA recovery in the 118 samples, the maximal variability of the data was estimated to be 0.62 log_10_ viral copy numbers per mite. The table provides an example with a mean copy number of 10^5^ viral copies per mite and the variations were calculated according to the convertion factor from viral copies per PCR to viral copies per mite. This variation in viral copy number remained within the tolerance limits of ± 1 log_10_ viral copies per mite. **Figure S2.** Accuracy profiles of the DWV-A and DWV-B quantitation methods. The assessment of the accuracy of the viral copy numbers quantified in individual bee heads by DWV-A and DWV-B RT-qPCRs was based on the construction and interpretation of accuracy profiles [17]. Crushed bee-head samples were spiked with pC1 and pFab1 recombinant plasmids including DWV-A or DWV-B VP3 coding sequence, respectively (recombinant plasmids used also for standard curves according to Schurr et al. [60] and processed for viral quantitation. Three plasmid load levels (10^6^, 10^8^ and 10^10^ copies/bee or head) were used to determine the standard deviation of reproducibility (SD_R_), the tolerance interval (± 2 × SD_R_), and the mean bias between the theoretical value and the mean of the obtained values of six results per load level. Both RT-qPCR methods quantified the recombinant plasmids within the limits of ± 1.0 log_10_ copies per bee head. The accuracy of DWV-B RT-qPCR was the lowest using spiked bee-head samples with 10^8^ plasmid copies/bee head. A and B, Quantification accuracy of DWV-A and DWV-B RT-qPCR methods, respectively. **Figure S3.** Raw viral copy numbers of *V. destructor* females from our in vitro study. Viral copy numbers were quantified in 9 mites from a naturally infested colony before inoculation. The mites were then transferred on artificially inoculated bee pupae for 4 days (post-inoc). The remaining mites were transferred onto untreated spinning larvae for 12 days until emergence of the bees (emergence). Bees and mites were maintained in an incubator (70% RH, 34.5°C). **Figure S4**. Boxplot showing the log_10_ transformed deformed wing virus-variant B (DWV-B) copy number in naturally infected bees, mite foundresses and mite offspring from the in situ study. The proportion of naturally infected individuals is shown on the* x*-axis. The diamonds indicate the mean value of the log_10_ transformed viral copy numbers.

## Data Availability

The data presented in this study are available on request from the corresponding author.
