# Supplementary material for: Transmission of deformed wing virus between Varroa destructor foundresses, mite offspring and infested honey bees
Source: Parasit Vectors. 2022 Sep 23;15:333. doi: 10.1186/s13071-022-05463-9 (PMC9502634; doi:10.1186/s13071-022-05463-9)
Supplement: Supplementary file 1 — Additional file 1: Text S1. Genome sequence of both DWV-A and DWV-B inocula used for the in vitro experiments. The NEBNext rRNA depletion kit (New England Biolabs; Ipwich, MA, USA; E6310) and NEBNext Ultra II directional RNA kit (New England Biolabs; E7760) were used to prepare the sequencing libraries. Libraries were pooled and sequenced with Nextseq 500 (Illumina Inc., San Diego, CA, USA) using the 150-cycle high-output kit at the sequencing core facility of the Brain & Spine Institute, Paris, France. Average coverage depth was used to calculate the number of read to sample in order to get an average coverage depth of 80×. Subsample reads were de novo assembled using Spades (V3.10.0; Bankevich et al. [77]) into contigs and aligned to the reference genome to generate a consensus sequence. We are grateful to Fabrice Sircoulomb (ANSES Sophia Antipolis), Pierrick Lucas and Yannick Blanchard (ANSES Ploufragan) for performing the sequence analysis. Table S1. Viral copy number quantified by RT-qPCR in inoculated emerging honey bees, viral inocula (calculated viral copy numbers according to the dilutions injected) and foundress mites used in the in vitro study. Table S2. Primers, probe and recombinant plasmids used for RT-qPCR quantification according to Schurr et al. [63]. Table S3. Factors used to convert the viral copy number per PCR into viral copy number per bee head or per mite. Figure S1. Distributions of V. destructor RNA amounts recovered after extraction, in relation to the stage of the mite considered. These variations in the total RNA amount were not taken into account because they had a limited impact on the general reproducibility of viral copy numbers quantified by RT-qPCR. Based on the ratios of RNA recovery in the 118 samples, the maximal variability of the data was estimated to be 0.62 log10 viral copy numbers per mite. The table provides an example with a mean copy number of 105 viral copies per mite and the variations were calculated according to the c [file 13071_2022_5463_MOESM1_ESM.docx]

**Additional files**

Dynamics of the transmission of *Deformed wing virus* between *Varroa destructor* foundresses, mite offspring and infested honey bees

Vincent Piou, Frank Schurr, Eric Dubois, and Angélique Vétillard

**Additional file: 1**

**Additional file 1: Text S1**

Genome sequence of both DWV-A and DWV-B inocula used for the *in vitro* experiments. The NEBNext rRNA depletion kit (New England Biolabs, E6310) and NEBNext Ultra II directional RNA kit (New England Biolabs; E7760) were used to prepare the sequencing libraries. Libraries were pooled and sequenced with Nextseq 500 (Illumina) using the 150-cycle high-output kit at the sequencing core facility of the Brain & Spine Institute, Paris, France. Average coverage depth was used to calculate the number of read to sample in order to get an average coverage depth of 80x. Subsample reads were de novo assembled using Spades (V3.10.0; Bankevich et al., 2012 [76]) into contigs and aligned to the reference genome to generate a consensus sequence. We are grateful to Fabrice Sircoulomb (ANSES Sophia Antipolis), Pierrick Lucas, and Yannick Blanchard (ANSES Ploufragan) for performing the sequence analysis.

**DWV-A sequence (H142):**

TATGCCTTCCATAGCGAATTACGGTGCAACTAACAATTTTAGATAGTAGCCATGAACAAACATTATAGTAGCTCACTACGTATTGATCATTTTTATAATGACTTGCGTAGCATGAAGCGCATGCTTGTAGTTGTAACTATGTTACTTTACAAGTTGGAGTTTACTATCTTGGATTATGAATATGTGCACTTAGTGTCTGTATTTATAGTCGTTTGTGGTTCAAGGTTTTGTGTTAGTAGTACACTTATGTATGAATGTACCTTTAGTATGAATGTTATAGAATGACAATATCGAAAGMAAAATCTTTATAAAATACAAAAATATTGTTTTCATTATTTCGATATGGTGTTTTATAGAGTAGATTGCCATGTGACCGCTCATAGAAGTCCATTATGGTTTATCAATCGAAGTTGAATGTATTTTATAAGGATATTATACTTAATTAGTAATATTAGTAGTCCGTAACTATTATCATCCTTTTTCAGTTTGATGTGATAATAGACCACTGCAGTATCGAGTAGAGTTTCGAATGCGTAGTGCAATAGTATAATCACTGTCACCGACCATCTATTGTAATGATAGATCTGTCGGAAACCATTATTTATGAAGTGACTAGCAATCATGGATTAAATTAGATGGTATTCTAGTTTAGAGGCGATTCGGCGCTGCGGTGCGACTGAAACTTCTAAATTGGCATGTCAGATTATATTATGAATGCGTTAGTAGTAATTTCTGCGATAGAGCTGGGACCCCTCAGTCTCTCAGGTATTGTACGAGGCGAAAGTGTGAAAGTTTTGTATGTATTTTTATATGTACGACTGTATCGGGAATTCCTTTAGCAAGAATCCTTTTAATACAGTATAATTTGTGCTACGGTACGTTACGTTCGCAGGGCACCCGTTAATGTCTCATAGCCCAGACGATGGCGGATGGAAAGACATCATATTTTATTTTAATGCTGTCTTTATTGCTGATTTATTTTGCTGTTTTTATTTGCTATTTTATATTTGCTAATTTTCATTATTGCTAAATATATTACATTGCTATTTTTATTGTATACGCTAGATTCAATTTTATTCTTTCTATATTTTCAATTTGATTTTGATTTCGAAGGTAAATATATATAATTAATTATTAAAAATGGCCTTTAGTTGCGGAACTCTTTCGTACTCTGCCGTCGCCCAAGCTCCGTCTGTCGCCCATGCACCTCGTACATGGGAAGTTGATGAAGCTAGGCGGCGCCGAGTTATTAAACGTTTGGCGTTGGAGCAAGAACGTATTCGTAACGTTCTTGACGTTGACGTCTATGCCCAGGCGACATGGGAACAGGAGGACGCGCGCGATAATGAGTTCCTAATGGAACAATTAAACAATTTATATACTATTTATTCGATCGCTGAACGTTGTACGCGTCGGCCTATCAAAGAGCACTCTCCTATATCAGTTTCGAATAGGTTTGCTCCACTGGAATCCCTTAAGGTCGAGGTCGGGCAAGAAGCAAGCGAATGTATATTTAAGAAACCTAAATATACGCGCGTTTGCAAGAAAGTGAAGCGTGTTGCAACTCGCTTCGTTCGTGAAAAAGTTGTTCGGCCTATGTGTTCTAGATCCCCTATGCTATTATTTAAGCTTAAGAAAGTTATTTATGATTTGCATTTATATAGATTAAGAAAACAGATTAGGATGTTGAGACGTCAAAAACAGCGCGATTATGAGTTAGAGTGTGTTACTAATCTGTTACAATTATCGAATCCGGTGCAGGCAAAACCAGAGATGGATAACCCTAATCCAGGACCTGATGGTGAGGGTGAAGTTGAATTAGAAAAGGATAGTAATGTTGTTTTAACAACTCAGCGAGATCCTAGCACATCCATTCCAGCGCCGGTGAGCGTAAAATGGAGTAGATGGACTAGTAATGATGTAGTAGATGATTACGCCACGATTACATCTCGATGGTATCAGATTGCTGAATTTGTTTGGTCGAAGGATGATCCATTTGATAAAGAGTTAGCACGCTTAATTTTGCCTCGAGCTTTGTTATCTAGTATAGAGGCTAATTCTGATGCTATATGTGATGTGCCTAATACTATCCCATTTAAGGTACACGCATATTGGCGAGGCGATATGGAAGTTAGAGTTCAAATTAATTCAAATAAATTCCAAGTTGGTCAATTACAAGCTACTTGGTATTATTCGGATCATGAGAACTTGAATATATCGTCTAAGAGAAGCGTTTATGGATTTTCACAAATGGATCATGCTTTGATTAGTGCGTCAGCAAGTAATGAAGCAAAATTAGTTATTCCATATAAGCATGTTTATCCATTTTTACCAACAAGAATTGTACCAGATTGGACTACTGGCATTTTAGATATGGGTGCTTTGAACATTCGTGTAATTGCTCCCTTACGGATGAGTGCTACTGGTCCAACTACCTGTAATGTCGTCGTGTTTATTAAATTAAATAACAGCGAGTTTACAGGGACTTCTTCTGGTAAATTTTATGCGAGCCAAATCAGGGCAAAACCTGAGATGGATCGTATATTAAATTTAGCAGAGGGATTGTTGAATAACACAATTGGCGGTAATAATATGGATAATCCTTCTTATCAACAATCTCCTCGTCATTTTGTCCCGACTGGTATGCACAGCTTAGCTTTAGGTACTAATTTAGTTGAACCATTACATGCATTACGTTTGGATGCAGCCGGTACGACACAACATCCTGTAGGTTGTGCTCCTGATGAAGATATGACTGTATCCTCCATTGCATCTCGATATGGATTAATTAGACGGGTACAATGGAAGAAAGATCATGCTAAAGGATCACTTTTGTTACAATTAGATGCTGATCCATTTGTGGAGCAAAGAATTGAGGGTACGAATCCAATATCCTTGTATTGGTTCGCACCCGTGGGTGTAGTATCTAGTATGTTTATGCAATGGCGTGGTTCATTAGAATATAGGTTTGATATTATAGCATCCCAATTTCATACTGGTAGGTTAATTGTAGGTTATGTGCCCGGTTTGACAGCATCTTTGCAACTTCAAATGGACTATATGAAATTGAAGTCATCGAGTTATGTAGTATTTGATTTACAAGAAAGTAATAGCTTCACTTTTGAGGTGCCATATGTTTCATATAGACCATGGTGGGTGCGTAAATATGGTGGCAATTATTTACCCTCGTCAACTGATGCTCCTAGTACACTATTTATGTATGTGCAGGTTCCGTTGATACCTATGGAAGCTGTTTCAGACACTATTGATATTAATGTGTACGTACGGGGCGGTAGCTCATTTGAAGTTTGCGTTCCAGTCCAACCTAGTTTAGGTTTGAATTGGAATACAGACTTTATTTTACGTAATGACGAAGAATATAGGGCTAAGACAGGTTATGCACCATATTATGCTGGAGTGTGGCATAGCTTCAATAATAGTAATTCTCTTGTTTTTAGGTGGGGATCTGCTTCTGATCAAATTGCTCAGTGGCCGACAATTTCAGTACCAAGAGGTGAGCTAGCTTTCTTACGAATTAAGGATGGAAAGCAAGCTGCTGTAGGAACTCAACCTTGGCGTACGATGGTTGTTTGGCCTTCTGGTCATGGTTATAATATTGGTATACCCACGTATAATGCTGAACGAGCTCGCCAGCTTGCACAACACTTATATGGTGGTGGATCATTAACTGATGAGAAGGCCAAACAATTATTTGTTCCTGCTAATCAACAAGGACCTGGTAAGATAAGTAATGGAAATCCGGTATGGGAAGTTATGCGTGCACCCTTGTCAACACAGCGTGCGCATATGCAAGATTTTGAATTTATTGAAGCTATTCCAGAAGGAGAGGAGTCTCGCAATACTACAGTCTTGGATACGACCACTACTTTACAGTCGAGTGGATTTGGTCGTGCCTTCTTTGGAGAAGCTTTTAATGATCTTAAAACGTTAATGCGACGATATCAATTATATGGTCAATTATTATTGTCCGTTACTACGGATAAGGATATTGATCATTGTATGTTTACCTTCCCTTGTTTACCACAAGGGTTAGCGTTAGACATCGGTTCTGCTGGTTCTCCACATGAAATCTTTAATAGATGTCGTGATGGTATTATACCATTAATTGCATCTGGATATAGATTTTATAGAGGAGATTTGCGTTATAAGATCGTTTTTCCAAGTAATGTTAATAGTAACATTTGGGTGCAACATCGACCGGATCGTAGACTGGAAGGATGGTCCGCGGCTAAGATTGTAAATTGTGATGCTGTGTCTACTGGTCAAGGGGTGTATAATCATGGTTATGCTAGTCACATTCAAATCACGCGTGTAAATAATGTTATAGAATTGGAAGTTCCATTTTATAATGCTACTTGTTATAATTATTTACAGGCGTTCAATGCGTCTAGCGCTGCATCTAGTTATGCAGTATCTTTAGGAGAAATATCGGTTGGTTTTCAAGCTACAAGTGATGATATTGCATCTATTGTTAACAAACCTGTTACTATTTATTATAGTATTGGAGATGGTATGCAATTTTCTCAGTGGGTTGGATATCAACCGATGATGATCCTAGACCAGCTTCCTGCACCAGTAGTAAGGGCCGTGCCTGAGGGCCCTATTGCGAAGATTAAAAACTTCTTCCATCAAACAGCCGATGAAGTTAGAGAAGCTCAGGCAGCAAAGATGCGTGAAGATATGGGTATGGTTGTCCAAGATGTTATTGGAGAACTTAGCCAGGCCATACCGGATCTTCAACAACCGGAGGTTCAAGCAAATGTCTTCTCACTGGTGTCTCAGTTAGTGCATGCTATTATAGGTACTAGTTTGAAGACAGTTGCTTGGGCGATTGTTTCGATTTTTGTGACTTTAGGATTAATTGGACGCGAAATGATGCATTCAGTTATTACTGTAGTCAAGCGGTTACTTGAGAAATATCACTTGGCGACGCAACCCCAGGAATCCGCCAGTTCAAGTACTGTTATATCTGCCGTTCCAGAAGCTCCCAATGCTGAAGCGGAGGAGGCAAGTGCTTGGGTATCCATTATTTATAATGGTGTGTGTAATATGCTTAATGTGGCTGCTCAAAAACCGAAACAATTTAAAGATTGGGTAAAATTAGCTACTGTAGATTTTAGTAATAATTGTAGAGGTAGTAATCAGGTATTTGTATTTTTCAAGAATACATTTGAAGTATTGAAGAAAATGTGGGGGTATGTATTTTGTCAGAGTAATCCTGCAGCGCGTTTGTTGAAAGCTGTGAATGACGAGCCTGAGATTTTGAAAGCATGGGTGAAGGAATGTCTGTATTTGGATGATCCCAAATTCAGAATGCGTCGAGCGCATGATCAAGAGTATATCGAGAGAGTGTTTGCGGCACATTCATATGGACAAATTTTGCTACATGATTTAACTGCTGAAATGAATCAATCACGAAATTTGAGTGTATTTACACGTGTGTATGATCAAATTTCAAAATTGAAGACCGATCTTATGGAAATGGGATCGAATCCATATATAAGGCGTGAATGTTTTACGATATGCATGTGTGGTGCATCTGGGATTGGAAAATCATATTTGACCGATTCTTTATGCAGCGAGCTCTTACGTGCGAGTCGTACTCCTGTGACAACAGGCATAAAATGTGTTGTTAATCCATTATCTGATTATTGGGATCAATGTGATTTTCAGCCTGTCTTGTGCGTTGACGACATGTGGAGTGTTGAAACATCTACTACGCTCGATAAGCAGTTGAATATGCTTTTCCAGGTTCATTCCCCTATCGTGCTTTCTCCTCCTAAAGCTGATTTAGAAGGTAAGAAGATGCGATATAACCCGGAAATATTCATATACAATACGAATAAACCTTTTCCAAGGTTTGATCGTATTGCTATGGAAGCTATTTATCGGCGTAGAAATGTTTTGATTGAGTGTAAAGCGAGTGAAGAGAAGAAGCGGGGATGCAAGCATTGTGAGGATGATATTCCTATTGCTGAATGTAGTCCTAAGATGTTGAAAGATTTCCATCATATTAAATTTAGGTATGCACATGATGTATGTAATTCCGAGACTACATGGTCTGAATGGATGACGTATAATGAATTCCTTGAATGGATAATTCCTGTGTATATGGCTAACCGTCGTAAGGCGAATGAATCGTTTAAAATGCGTGTAGATGAAATGCAAATGTTACGTATGGATGAACCATTGGAAGGTGATAATATTCTCAATAAGTATGTTGAAGTTAATCAGCGCTTAGTCGAGGAAATGAAGGCATTTAAGGAGCGTACGCTATGGTCAGATTTACATCGCGTAGGTGCTGAAATTAGTGCGTCAGTTAAGAAAGCCTTACCAACCATTTCCATAACCGAAAAACTACCACATTGGACTGTTCAATGTGGTATTGCTAAACCTGAGATGGATCATGCTTATGAGGTTATGAGTTCGTATGCAGCTGGAATGAATGCAGAGATTGAAGCACATGAACAAGTTCGGCGTTCATCAGTGGAATGTCAATATACAGAGCCTCAAGCTCCAAGAAATCCTGATGATGAAGGGCCAACCATAGATGAAGAACTTATGGGTGACACTGAATTTACATCACAGGCTCTAGAACGTCTTGTGGATGAAGGTTATATAACTGGAAAACAGAAGAAATATATGGCTACGTGGTGTAGTAAGCGTCATGAACATATTGCTGACTTTGATCTAGTGTGGACTGATAATTTGCGTGTGTTAAGTGCGTATGCACATGAACGCTCATCTTCAACTCGACTTTCTACGGATGACGTTAAGTTATATAAAACAATTAGCATGTTACATCAAAAGTATGATACCACAGAGTGTGCTAAATGTCAACATTGGTATGCTCCGTTGACTGATATTTATGTTGATGACAAGAAATTGTTCTGGTGTCAGAAAGAGAAAAAGACACTTATTGACGTCCGCAAATTGTCGAAAGAAGATGTGACTGTTCAATCAAAATTGATTAATTTATCTGTTCCTTGTGGTGAAGTGTGTATGTTACATTCAAAATATTTCAATTATCTTTTCCACAAAGCATGGTTGTTTGAGAACCCAACTTGGCGTCTAATATATAATGGTACCAAGAAGGGTATGCCTGAGTACTTTATGAATTGTGTGGATGAAATTTCATTAGATTCTAAATTTGGTAAAGTGAAAGTGTGGTTGCAAGCGATCATTGATAAGTATTTAACTCGTCCCGTGAAAATGATTCGTGATTTTCTTTTTAAGTGGTGGCCGCAAGTTGCGTATGTGTTGAGCTTGCTTGGTATAATTGGTATAACTGCGTATGAAATGAGAAATCCGAAACCAACTTCTGAGGAATTAGCTGATCATTATGTGAATAGGCATTGTAGCTCTGATTTTTGGTCACCAGGACTGGCATCACCTCAAGGATTGAAATATAGTGAAGCAGTGACAGTAAAGGCACCTAGAATCCATAGATTGCCAGTTACTACTAAGCCTCAGGGATCAACACAACAAGTAGACGCTGCTGTGAATAAAATTCTACATAACATGGTTTATATTGGTGTTGTTTTCCCAAAAGTGCCTGGTAGTAAGTGGCGAGATATTAATTTTAGGTGTCTTATGCTTCATAATAGGCAATGTTTAATGTTGAGGCATTATATTGAGTCAACTGCCGCCTTTCCTGAGGGAACTAAGTACTATTTTAAGTATATTCATAATCAAGAGACTAGAATGTCTGGTGATATTTCTGGTATTGAAATTGATTTGTTGAATTTACCTAGATTGTATTATGGTGGTCTCGCGGGAGAGGAGTCATTTGATAGTAATATTGTGCTTGTGACTATGCCTAATCGTATTCCTGAGTGTAAGAGCATTATTAAATTTATAGCGTCACATAATGAGCATATACGTGCTCAGAATGATGGAGTGTTAGTAACTGGTGACCATACTCAGTTATTGGCTTTCGAGAATAATAATAAAACTCCAATAAGTATCAACGCTGATGGTTTGTATGAGGTCATACTTCAAGGAGTATATACCTATCCATATCATGGCGATGGTGTTTGTGGTTCGATATTGTTGTCTCGGAATTTACAACGGCCAATTATAGGTATCCATGTTGCTGGTACTGAAGGATTGCATGGCTTTGGAGTTGCTGAACCGCTTGTACATGAAATGTTTACCGGTAAAGCAATTGAGAGTGAAAGAGAGCCGTATGATCGTGTGTATGAACTTCCGTTGCGTGAATTGGATGAATCTGATATTGGTTTAGATACTGATTTATATCCGATTGGTAGAGTGGATGCAAAGCTAGCTCATGCTCAAAGCCCTTCTACTGGGATTAAAAAGACGCTTATCCATGGAACATTTGATGTAAGGACTGAACCAAATCCGATGTCATCACGTGATCCAAGAATAGCGCCACATGATCCTTTGAAGTTAGGGTGTGAAAAGCATGGCATGCCTTGTTCACCGTTTAATAGAAAACATCTGGAATTAGCGACAAATCATTTGAAAGAAAAATTAGTTTCAGTAGTTAAACCAATAAATGGTTGCAAGATTAGAAGCTTGCAAGATGCTGTATGTGGTGTGCCTGGTTTAGACGGTTTTGATTCGATATCTTGGAATACTAGTGCTGGTTTTCCTTTGTCTTCATTAAAGCCACCTGGAACATCAGGTAAGCGATGGTTGTTTGATATTGAGCTACAAGACTCGGGATGTTATCTCTTGCGTGGAATGCGTCCCGAACTTGAGATTCAATTATCAACGACACAGTTAATGAGGAAAAAGGGAATAAAACCTCACACTATATTCACGGATTGTTTGAAAGATACCTGTTTGCCTGTTGAAAAATGTAGAATACCTGGTAAGACTAGAATATTTAGTATAAGTCCGGTACAGTTTACCATACCGTTTCGACAGTATTACTTAGACTTTATGGCATCCTATCGAGCTGCACGACTTAATGCTGAGCATGGTATTGGTATTGATGTTAACAGCTTAGAGTGGACAAATTTGGCAACAAGGTTGTCTAAGTATGGCACTCACATCGTGACAGGAGACTATAAGAATTTTGGTCCTGGGTTAGATTCCGATGTTGCAGCTTCAGCGTTCGAAATTATTATCGACTGGGTATTACATTACACCGAAGAAGATAATAAAGACGAAATGAAGCGAGTAATGTGGACCATGGCGCAAGAGATATTAGCGCCTAGTCATCTATATCGCGATTTGGTATACCGAGTGCCTTGTGGAATTCCATCCGGTTCTCCAATAACGGACATATTGAACACGATTTCAAATTGTTTGTTAATTAGGTTAGCTTGGTTAGGTATTACTGACTTGCCTTTGTCCGAGTTCTCTCAAAATGTTGTTCTTGTCTGTTATGGCGACGATCTTATCATGAATGTTAGCGATAACATGATTGATAAGTTTAATGCCGTGACGATAGGAAAATTCTTTTCTCAATATAAGATGGAATTTACGGATCAGGATAAGTCAGGAAATACTGTAAAGTGGCGGACGTTACAGACTGCTACTTTCTTAAAACACGGGTTCTTAAAACATCCAACTAGACCTGTGTTTCTAGCTAACCTGGACAAGGTCTCGGTAGAAGGAACGACGAATTGGACTCATGCTCGAGGATTGGGTCGTCGTACAGCAACCATAGAAAATGCTAAACAAGCGTTAGAATTAGCATTTGGGTGGGGTCCTGAATATTTTAATTATGTCAGAAATACCATTAAAATGGCTTTTGACAAGTTGGGTATTTATGAGGACCTTATCACATGGGAAGAAATGGATGTCAGATGTTACGCTAGTGCGTAATATTAAATTTTTAAATACTCATTAATTTTAATTTTATTTTAGGTTATTGGAATTGAGGGAAGTACCACCCCCCAAGACCTTCGTTTTAAATCTACTAAGAGGAGTAAACCTATATATAAGAGTCTAAAGACAGAGTGGATTAGACCATCATCTTTAGCTTATATATGGGGAAGGTTGAGTTGCCTCTAAAGACTCAGCTCCATAGTAGAGTAGTTTTAATTACGATTAAAGTGGTACTCTAGGTTAGGTGTTACTCGCGTATTATCAATTAGTGGTAATGCGTCCTAATTTTAGTATAGTTTTAACCATAATAGT

**DWV-B sequence (H122):**

CATAGCGAATTACGGTGCAACTAACAATTTTAGATAGTAGCCATGAACAAACATTATGATTACTCACTACGTATTGATCATTTTTATAATGACTTGCGTAGCATGAAGCGCATGCTTGTAGTTATAACTATGTTATTTTGCAAGTTGGAGATAATTGTATTGGATTATGGATGCGTGCACTAAGTGTCTACATCTATAGTCGTTTGTGGTTCAAGTTTTTGTGTTAGTAGTACAATCTTGAAGAATGTAAGTATCGTATGAATGATATTTGAATGACAACACTGAAGTATAAAATATATAAAATCTAAAAATATTTTTAATCTTATTCAGTGTAGTGTTTGATAGAGTAGAATGCCATGTGACCGCTCAAAGAAGTCCATTATGGTATATCATTCGAAGTCGAATACTTGTGTATAGTTATTGTATTTTATTAGTAATATTAGTAGTCCGTAACTATCATAATCCTATTATAGTTTGATTATATGATAGACCACTGCAGTATCGAGTAGAGTTTAGAAAGAGTAGTGCAATAGTAAGATCACTGTCACCGACCACTCATTGTAATAGTGAGGTTTGTCGGAAACCAATTATTGTGCAGCGACTAGCAATCGTGAATCAATATAGTTGGTATTCTAAATATGAGACGATTCGGCGATTTTATTGCGACTGAAATTTCATATTTAGCATGTCAGGTCTTATTATGAATGCTCGAGTATTTATTTCTGCGGTAGAGTAGGGACCCCTCTATCTCTCAGGTACTGTATGAGGCGAAAGTGTGAAAGTAATTTATGTCTCTATACATAAGTGACTGTATTGGGATTTCCTTTGGCAAGAATCCTTTTAATACAGTATAATTTATGCCACGGTACGTTACGTTCGCAGGGCACCCGTTAATGTCACATAGTCCAGACGATGACGAATGGAAAGACATTACTTTTTATTTTAATGCTACGATTATTGCTGTTTTATTTTGCTGTTTTTATTTGTTATTATATTTTGCTATTTTATTATTGCTAAATATATTTCTTTGCTATTTTTGTTTTATATATTAGATTCAATTCTTTTTATTTTATATTTTCAATTTGATTTTGATTTTGAAGGTAAATATATATAAAAATGGCATTTAGTTGTGGAACTCTTTCTTATGCTGCTGTTGCCCAAGCTCCCTCTGTAGCTCATGCTCCCCGTAGTTGGGAGATTGATGAAGCTAGGCGTCGACGCGTTATTAAGCGTTTGGCGTTGGAACAGGAACGGATTCGAAACGTTCTTGATGTCACTGTGTATGATCATACAACGTGGGAGCAAGAAGATGCGCGTGATAATGAGTTCCTTACGGAACAATTGAATAATTTATATACGATATATTCTATAGCTGAAAGATGTATCCGCCGCCCTGTTCAAGAACATGTCCCCATTTCAATCAGTAATAGATATTCCCCTTTAGAATCCCTTAAGATTGAGGTAGGAAAAGACGCGGGTGAGTTCGTATTTAAGAAACCCAAATATACAAAGATTTGTAAGAAAGTGAAACGAGTGGCATCAAAATTTGTGCGCGAGAAAGTTGTTAGGCCCGTTTGTAATCGATCGCCCATGTTATTATTTAAAATTAAGAAAGTAATATATGATTTACATTTGTATCGGTTACGGAAACAAGTTCGGCTTCTCAGACGCGAAAAACAGCGTGAATACGAGTTAGAGTGTGTTACTAGTTTGCTACAACTATCTAATCCTGTTTCGGCTAAACCTGAGATGGACAATCCTAATCCTGGTCCAGATGGTGAAGGTGAAGTTGAATTAGAAAAGGATAGTAATGTAGTATTAACTACACAACGTGATCCTAGTACCTCTATTCCTGCTCCAACTAGTGTGAAGTGGAGTAGATGGACTAGTAATGATGTTGTGGATGATTATGCCACTATAACTTCGCGTTGGTATCAAATTGCCGAATTTGTGTGGTCAAAGGATGATCCATTTGATAAGGAATTGGCGCGTTTAATTTTACCTCGAGCTTTGTTATCTAGTATTGAGGCTAATTCTGACGCTATTTGTGATGTACCTAATACTATTCCGTTTAAGGTACATGCATATTGGCGTGGAGATATGGAAGTTCGAGTGCAGATTAACTCGAATAAATTCCAGGTTGGCCAATTACAGGCAACTTGGTACTATTCGGATCATGAAAATTTGAATATTCAGACGAAGCGAAGTGTGTATGGTTTTTCGCATATGGATCATGCTTTGATTAGCGCATCAGCGAGTAATGAAGCAAAATTAGTGATACCTTTTAAACACGTATATCCATTCTTACCAACGCGTGTCGTTCCTGATTGGACAACTGGTATTCTTGATATGGGTACCTTAAATATTCGTGTAATTGCACCACTACGTATGAGTGCGACGGGACCAACCACTTGTAATGTTGTAGTATTTATTAAGTTAAATAATAGTGAATTCACTGGCACTTCTTCTGGTAAGTTTTACGCGAATCAAATTAGGGCAAAACCTGAAATGGACCGTGTGTTAAATTTGGCAGAAGGATTACTAAATAATACTGTAGGTGGTTGTAATATGGATAATCCGTCATATCAGCAATCTCCGCGTCATTTTGTTCCTACTGGTATGCATAGTTTAGCTTTAGGCACTAATTTAGTAGAGCCTTTGCATGCATTACGATTAGACGCATCAGGTACAACACAACATCCAGTTGGGTGTGCGCCTGATGAAGATATGACCGTATCTTCTATTGCATCACGATATGGTTTAATTCGCCAAGTGCAATGGAAGAAAGACCATGCGAAAGGATCGTTATTACTACAACTTGATGCTGATCCTTTCGTTGAACAGAAAATTGAGGGAACCAATCCAATTTCTTTGTATTGGTTTGCTCCGGTTGGAGTCGTATCTAGTATGTTTATGCAATGGAGAGGTTCTTTAGAATATAGATTTGATATTATAGCATCCCAATTTCATACGGGTAGGTTAATTGTAGGTTATGTTCCTGGACTGACTGCCTCTTTACAACGTCAAATGGACTATATGAAATTGAAGTCATCTAGTTATGTGGTGTTTGACTTACAGGAAAGTAATAGTTTTACGTTTGAAGTGCCCTATGTGTCATACAGACCGTGGTGGGTGCGTAAGTATGGTGGTAATTATCTGCCATCTTCTACTGATGCGCCTAGCACACTGTTTATGTATGTACAAGTACCATTGATACCTATGGAAGCTGTTTCTGATACTATAGATATCAATGTGTATGTGCGTGGTGGCAGTTCGTTTGAGGTTTGTGTTCCAGTCCAACCTAGTTTAGGTTTGAACTGGAATACAGATTTCATATTACGTAATGATGAGGAGTACCGCGCAAAGAATGGATATGCACCATATTATGCTGGTGTGTGGCATAGCTTCAATAATAGTAATTCGCTTGTTTTTAGATGGGGTTCGGCTGCAGATCAAATTGCTCAATGGCCAACAATAACAGTGCCTCGAGGAGAGTTGGCATTCCTGCGTATCCGCGATGCTAAGCAAGCTGCTGTAGGAACGCAACCTTGGCGTACTATGGTTGTTTGGCCTTCAGGTCATGGATATAATATTGGAATACCAACTTATAATGCTGAACGAGCAAGACAACTTGCTCAGCATTTGTATGGTGGTGGGTCTTTGACAGATGAGAAGGCTAAGCAATTATTTGTGCCTGCTAACCAGCAAGGACCCGGCAAAGTAAGTAACGGTAACCCCGTCTGGGAAGTAATGCGCGCGCCTCTTGCAACTCAGCAAGCGCATATACAAGATTTTGAATTTGTTGAAGCTGTTCCAGAAGGCGAAGAATCACGCAACACTACGGTGCTAGACACGACAACAACGTTACAGTCTAGTGGATTTGGTCGCGCTTTCTTCGGTGAGGCATTTAACGATCTTAAGACGTTAATGCGCCGATACCAATTATATGGTCAATTATTGTTATCCGTTACTACGGATAAGGATATTGATCATTGTATGTTTACCTTCCCTTGTTTACCTCAAGGGTTAGCGTTAGATATAGGTTCGGCTGGATCTCCTCATGAAATATTTAATCGCTGCCGTGATGGTATCATTCCATTGATAGCGTCAGGGTATCGGTTTTATCGAGGCGATTTACGGTTCAAAATTGTTTTCCCAAGTAACGTTAATAGCAATATTTGGGTACAACATCGACCAGATCGTAGACTGAAAGGATGGTCTGAAGCGAAAATAATAAACTGTGATGCTGTATCTACTGGACAAGGTGTTTATAATCATGGATATGCTAGTCATATTCAGATTACGCGTGTAAATAATGTTATAGAATTGGAAGTCCCGTTTTATAACGCTACGTGCTATAATTATTTGCAAGCGTTTAATCCATCTAGTGCTGCGTCGAGTTATGCCGTTTCGCTCGGAGAGATTTCGGTTGGTTTTCAGGCTACTAGTGATGACATTGCAGCCATAGTTAATAAACCTGTAACTATATATTACAGTATTGGCGATGGTATGCAGTTTTCGCAGTGGGTTGGTTATCAACCAATGATGATTCTAGATCAATTGCCAGCACCAGTAGTTAGGGCTGTGCCTGAGGGCCCTATAGCGAAGATAAAGAACTTTTTCCACCAAACGGCAGATGAAGTTCGAGAAGCTCAGGCCGCAAAGATGCGTGAAGATATGGGTATAGTAGTCCAAGACGTTATAGGAGAGTTAAGTCAGGCTATACCCGATCTTCAACAACCGGAAGTTCAAGCGAATGTTTTTTCTCTGGTGTCACAGTTAGTGCATGCTATCATCGGTACTAGTCTTAAAACAGTTGCTTGGGCGATTGTTTCGATTTTTGTAACTTTAGGTTTGATTGGACGTGAAATGATGCATTCAGTTATAACTGTAGTTAAGCGGTTATTAGAAAAATATCACTTGGCGACGCAACCCCAGGAATCCGCCAATTCAGGTACGGTTATTTCTGCTATTCCAGAAGCACCCAATGCTGAAGCAGAGGAAGCCAGTGCCTGGGTATCCATTATTTATAATGGTGTGTGTAATATGTTGAATGTAGCCGCTCAAAAACCGAAACAATTTAAAGATTGGGTAAAATTAGCTACCGTAGATTTTAGTAATAATTGTAGAGGTAGTAATCAGGTATTTGTGTTTTTCAAGAATACGTTTGAAGTGTTGAAGAAAATGTGGGGTTATGTGTTTTGTCAGAGTAATCCTGCAGCGCGACTCTTGAAAGCAGTGAATGATGAACCTGAGATTTTAAAAGCGTGGGTTAAAGAATGTCTGTATTTAGATGATCCTAAATTTAGAATGCGACGTGCGCATGATCAAGAGTATATTGAGAGAGTGTTTGCGGCCCATTCGTATGGACAAATTTTATTGCATGACTTAACGGCTGAAATGAATCAATCGCGTAATTTAAGTGTGTTTACGAGAGTGTATGATCAAATATCTAAATTGAAGACGGATCTCATGGAAATGGGATCAAACCCATATATCAGGCGTGAATGCTTTACGATTTGTATGTGTGGTGCATCTGGAATTGGTAAGTCTTATTTAACTGATTCTTTATGCAGCGAGCTCTTACGTGCGAGTCGTACTCCAGTGACAACGGGCATTAAGTGTGTCGTGAACCCTTTGTCTGATTATTGGGATCAGTGTGATTTTCAGCCCGTTTTATGTGTTGATGACATGTGGAGTGTTGAAACGTCTACTACGCTCGATAAACAGTTAAACATGCTATTTCAGGTTCATTCACCAATTGTACTTTCACCTCCTAAAGCTGATTTAGAAGGTAAGAAAATGCGTTATAATCCTGAAATATTCATATATAATACGAATAAACCTTTTCCGAGGTTTGATCGTATAGCTATGGAAGCTATTTATCGACGTAGAAACGTTTTAATTGAATGTAAGGCTAATGAAGAGAAGAAGCGTGGATGTAAACATTGTGAGAATAATATACCCATTGCTGAATGTAGTCCAAAAATTTTGAAAGATTTTCATCACATTAAATTTCGTTATGCTCATGATGTGTGTAATTCTGAAACTACGTGGTCTGAGTGGATGTCGTATAATGAATTTTTGGAATGGATTACTCCTGTATATATGGCTAATCGACGTAAAGCAAATGAATCGTTTAAGATGCGTGTTGATGAAATGCAAATGTTGCGTATGGATGAGCCCTTGGAAGGCGATAATATTTTAAATAAGTATGTTGAAGTTAATCAGCGCTTAGTTGAGGAAATGAAAGCTTTTAAAGAGCGAACCCTCTGGGCTGATTTACAACGTGTTGGCTCAGAGATTAGTACTTCAGTTAAGAAAGCATTACCAACTATTTCCATTACTGAGAAGCTACCACATTGGACTATCCAATGTGGCATAGCTAAGCCTGAAATGGATCATGCTTATGAAGTTATGAGTTCATATGCAGCAGGAATGAACGCGGAAATTGAAGCGCATGAACAAGTTCGTCGTTCTTCTTTGGAATGTCAGTACATTGAGCCTTCAACTTCAAGACCTCTGGATGAAGAGGGTCCTACTATCGACGAGGAATTACTTGGCGAAGTAGAATTTACTTCTTCAGCTTTGGAGCGTTTGGTTGATGAGGGGTATATTACTGGTAAACAAAAGAAGTATATGGCAACTTGGTGTACGAAACGAAGAGAGCATGTATCCGATTTTGATTTAGTATGGACGGATAATTTGCGTGTTTTGAGTGCGTATGTCCACGAGCGTTCTACATCTACGCGTTTATCTACCGATGATGTTAAATTATTTAAGACGATTAGTATGTTACATCAGAGGTATGATACCACTGATTGTGCAAAATGCCAACATTGGTATGCACCATTAACAGCTATTTATGTTGATGATAGAAAGCTATTTTGGTGCCAGAAGGAGACTAAAACTTTGATAGATGTTCGTAAATTGTCGAAAGAGGACGTTACAGTCCAATCGAAATTAATTAACTTATCGGTTCCGTGCGGCGATGTATGTATATTACATTCTAAGTACTTTAATTATTTATTCCATAAAGCGTGGTTGTTTGAAAATCCAACATGGCGTTTAATATATAATGGTACTAAGAAAGGTATGCCTGAGTATTTCATGAATTGCGTGGATGAAATTTCATTAGATTCAAAATTTTGTAAAGTAAAGGTTTGGCTTCAAGCAATTATTGATAAATATTTGACTCGTCCAGTGAAAATGATTCGTGACTTTCTATTTAAATGGTGGCCGCAAGTAGCATATGTGTTAAGTTTGTTAGGTATAATTGGTATAACTGCGTATGAGATGCGTAATCCTAAATCAACAGCAGAAGACTTGGCTGAGCACTATGTTAATAGGCATTGTAGTTCAGATTTTTGGTCACCAGGTATGGCGACTCCCCAGGGATTAAAATATAGTGAAGCGATAACAGCTAAAGCGCCTAGAATCCATAGATTGCCCGTTACTACTAGACCTCAGGGATCAACGCAACAAGTTGACGCCGCTGTGAATAAGATTTTGCAGAATATGGTGTATATCGGTGTAGTGTTTCCGAAAGTGCCTGGTAGTAAGTGGCGAGATATTAATTTTAGATGTCTTATGCTCCATAATCGGCAATGTTTGATGTTGCGGCATTACATTGAGTCGACGGCTGCTTTTCCGGAGGGTACCAAATACTATTTTAAGTATATTCATAATCAAGAAACTCGAATGTCAGGTGATATATCTGGTATTGAGATTGATTTATTGAGTTTACCTAGATTGTATTATGGTGGCTTAGCTGGGGAAGAGTCGTTCGATAGCAATATAGTGTTAGTAACTATGCCGAATAGAATTCCTGAGTGTAAGAGTATTGTGAAGTTTATAGCTTCACATGCTGAACATGCTCGTGCTCAAAATGATGGTGTGTTAGTTACTGGTGAACATACTCAGTTATTGGCGTTCGAGAATAATAATAAAACACCTATAAGTATTAATGCTGATGGTTTGTATGAGGTTATACTTCAAGGAGTATACACTTATCCATACCATGGTGATGGTGTTTGTGGGTCTATATTATTGTCTCGTAATTTACAACGACCAATTATAGGAATCCATGTAGCTGGTACTGAAGGATTACATGGCTTTGGTGTTGCTGAACCTCTTGTTCATGAGATGTTCACTGGGAAAGCAATAGAGAGTGAAAGGGAACCGTATGATCGTGTGTATGAATTACCTTTGCGTGAATTAGATGAATCTGATATAGGTTTGGATACTGATTTATATCCTATAGGAAGAGTTGATGCGAAATTAGCTCATGCTCAAAGTCCTTCAACAGGAATTAAAAAGACGCTTATTCATGGTACTTTTGATGTTCGGACTGAACCGAATCCGATGTCATCACGAGACCCAAGAATAGCGCCACATGATCCGTTGAAGTTAGGGTGTGAGAAACATGGTATGCCATGTTCTCCATTTAATCGAAAACATTTGGAATTAGCAACAACTCATTTAAAGGAGAAGTTAATTTCCGTAGTTAAACCTATAAACGGATGCAAGATTAGAAGTTTGCAAGATGCTGTGTGTGGTGTACCGGGTTTGGATGGCTTTGATTCAATATCCTGGAATACTAGTGCTGGTTTTCCTTTATCTTCATTAAAACCGCCAGGCTCTTCTGGTAAGCGATGGTTGTTTGATATTGAATTACAAGATTCAGGATGTTATCTTTTGAGAGGGATGAGACCTGAACTTGAGATACAGTTGACAACAACTCAGTTAATGAGGAAGAAGGGAATAAAGCCTCACACTATATTCACGGATTGTTTGAAAGATACATGTTTGCCTGTGGAAAAATGCAGAATACCTGGTAAGACTAGAATATTTAGTATAAGTCCCGTCCAATTTACGATTCCATTCCGACAATACTATCTCGATTTTATGGCGTCGTACCGTGCCGCTAGACTTAATGCTGAGCATGGAATAGGTATAGACGTGAACAGCTTGGAATGGACAAACTTGGCAACAAGTCTGTCGAAGTATGGCACGCATATTGTGACAGGAGATTACAAGAATTTTGGTCCTGGGTTAGATTCTGATGTTGCCGCTTCAGCTTTCGAAATTATCATTGATTGGGTGTTAAATTACACTGAAGAAGATGATAAAGACGAAATGAAGCGTGTAATGTGGACTATGGCTCAGGAAATCTTAGCTCCTAGTCACTTATGTCGTGATTTAGTATATCGCGTACCATGCGGTATTCCTTCTGGATCACCAATTACGGACATTTTGAATACTATTTCGAATTGTTTGTTAATTCGATTGGCTTGGCAAGGTATTACTGATTTGCCTTTATCCGAATTTTCTAGACATGTCGTGCTAGTTTGTTACGGTGATGATCTCATCATGAATGTAAGTGGTGAGATGATAGACAAATTCAACGCTGTAACAATTGGCGATTTCTTTTCGCGATATAAGATGGAATTTACGGATCAGGATAAATCTGGAAATACAGTGCGGTGGCGAACTTTACAAACTGCCACGTTTTTGAAGCATGGGTTCTTGAAACATCCAACAAGACCCGTGTTTCTAGCCAATCTGGATAAGGTTTCTATAGAAGGAACAACCAATTGGACACATGCTCGAGGATTGGGTCGTCGAGTAGCAACCATTGAGAATGCTAAACAAGCGCTAGAGTTGGCATTCGGATGGGGTCCCGAATACTTTAATCATGTTCGGAATACCATTAAAATGGCATTCGACAAGTTAGGTATTTATGAGGATCTCATCACATGGGAAGAAATGGATGTTAGATGTTATGCTAGCGCGTAATTTTAAGATTTTAATACTCATTAAAATTAATTTATATTTAGGTTATTGGAATTGAGGGAAGTACCACCCCCCAAGACCTTCGTTTTAAATCTACTAAGAGGAGTGAACTTGCATATAAGAGTCTAAAAGCAGAGTGGATTAGACCACCACTTTTAGCTTATATGTGAGGAAGGTTGAGTTGCCTCTAAAGACTCAGCTCCGTAGTAGAGTAGTTTTAGTTACGATTAAAGTGGTACTCTAGGTTAGGTGTTACTCGCGTATTGTCGCATAACGGCAATGCGTCCTAATTTTAGTATAGTTTAACCATAATAGG

**Additional file tables**

**Additional file 1: Table S1**. Viral copy number quantified by RT-qPCR in inoculated emerging honey bees, viral inocula (calculated viral copy numbers according to the dilutions injected), and foundress mites used in the *in vitro* study.

| **Sample** | **Viral copy number^a,b^** | | | | | |
| --- | --- | --- | --- | --- | --- | --- |
|  | **DWV-A** | **DWV-B** | **ABPV** | **BQCV** | **CBPV** | **SBV** |
| Emerging bees  (copies/bee) PB | 0 | 5.3 x 10^9^ | 3.5 x 10^1^  (2 positive/7) | 0 | 0 | 0 |
| Emerging bees  (copies/bee) DWV-A | 1.5 x 10^10^ | 2.9 x 10^8^ | n.d | n.d | n.d | 1.6 x 10^2^  (1 positive/7) |
| Emerging bees  (copies/bee) DWV-B | 1.4 x 10^1^ | 4.0 x 10^9^ | n.d | n.d | n.d | 5.6 x 10^2^  (3 positive/7) |
| DWV-A inoculum  (copies/2 µl) | 5.0 x 10^2^ | 4.5 x 10^-5^ | 0 | 0 | 0 | 0.9 x 10^1^ |
| DWV-B inoculum (copies/2µl) | 0 | 5.0 x 10^2^ | 0 | 0 | 0 | 4.9 x 10^1^ |
| Varroa foundress  (copies/mite) | 0 | 1.0 x 10^4^  (5 positives/9) | n.d | n.d | n.d | n.d |

a. 0: undetected

b. n.d: not done

**Additional file 1: Table S2.** Primers, probe and recombinant plasmids used for RT-qPCR quantification according to Schurr et al., 2019 [62]

| **Virus** | **Forward primer (5’ -> 3’)** | **Reverse primer (5’ -> 3’)** | **Probe (5’ -> 3’)** | **Recombinant plasmid** | **Reference** |
| --- | --- | --- | --- | --- | --- |
| ABPV | CATATTGGCGAGCCACTATG | CTACCAGGTTCAAAGAAAATTTC | (6-Fam)ATAGTTAAAACAGC TTTTCACACTGG(Tamra) | pB2 | Bakonyi et al., 2002 [78] |
| BQCV | GGTGCGGGAGATGATATGGA | GCCGTCTGAGATGCATGAATAC | (6-Fam)TTTCCATCTTTATCG GTACGCCGCC(Tamra) | pNC1-4 | Benjeddou et al. 2001 [79] |
| CBPV | CGCAAGTACGCCTTGATAAAGAAC | ACTACTAGAAACTCGTCGCTTCG | (6-Fam)TCAAGAACGAGACC ACCGCCAAGTTC(Tamra) | pAb2 | Blanchard et al. 2007 [80] |
| DWV-A | GCGGCTAAGATTGTAAATTG | GTGACTAGCATAACCATGATTA | (6-Fam)CCTTGACCAGTAGA CACAGCATC(Tamra) | pC1 | Blanchard et al. 2007 [80] |
| DWV-B | GGTCTGAAGCGAAAATAG | CTAGCATATCCATGATTATAAAC | (6-Fam)CCTTGTCCAGTAGA TACAGCATCACA(Tamra) | pFab1 | Grabensteiner et al. 2001 [81] |
| SBV | AACGTCCACTACACCGAAATGTC | ACACTGCGCGTCTAACATTCC | (6-Fam)TGATGAGAGTGGAC GAAGA(MGB) | pD1 | Schurr et al. 2019 [63] |

**Additional file 1: Table S3.** Factors used to convert the viral copy number per PCR into viral copy number per bee head or per mite

| **Bee protocol** | **Total volume** | **Tested volume** | **Factor** |
| --- | --- | --- | --- |
| Bee homogenate (1 bee head/0.5 ml) | 500 µl | 140 µl | 3.57 |
| Purified RNA | 60 µl | 11 µl | 5.46 |
| cDNA | 20 µl | 5 µl | 4.00 |
| **Conversion factor** | **77.92** | | |
|  |  | | |
| **Mite protocol** | **Total volume** | **Tested volume** | **Factor** |
| Mite homogenate (1 mite/50 µl) | 50 µl | 50 µl | 1 |
| Purified RNA | 60 µl | 11 µl | 5.45 |
| cDNA | 20 µl | 5 µl | 4.00 |
| **Conversion factor** | **21.82** | | |

**Supplementary figures**

**
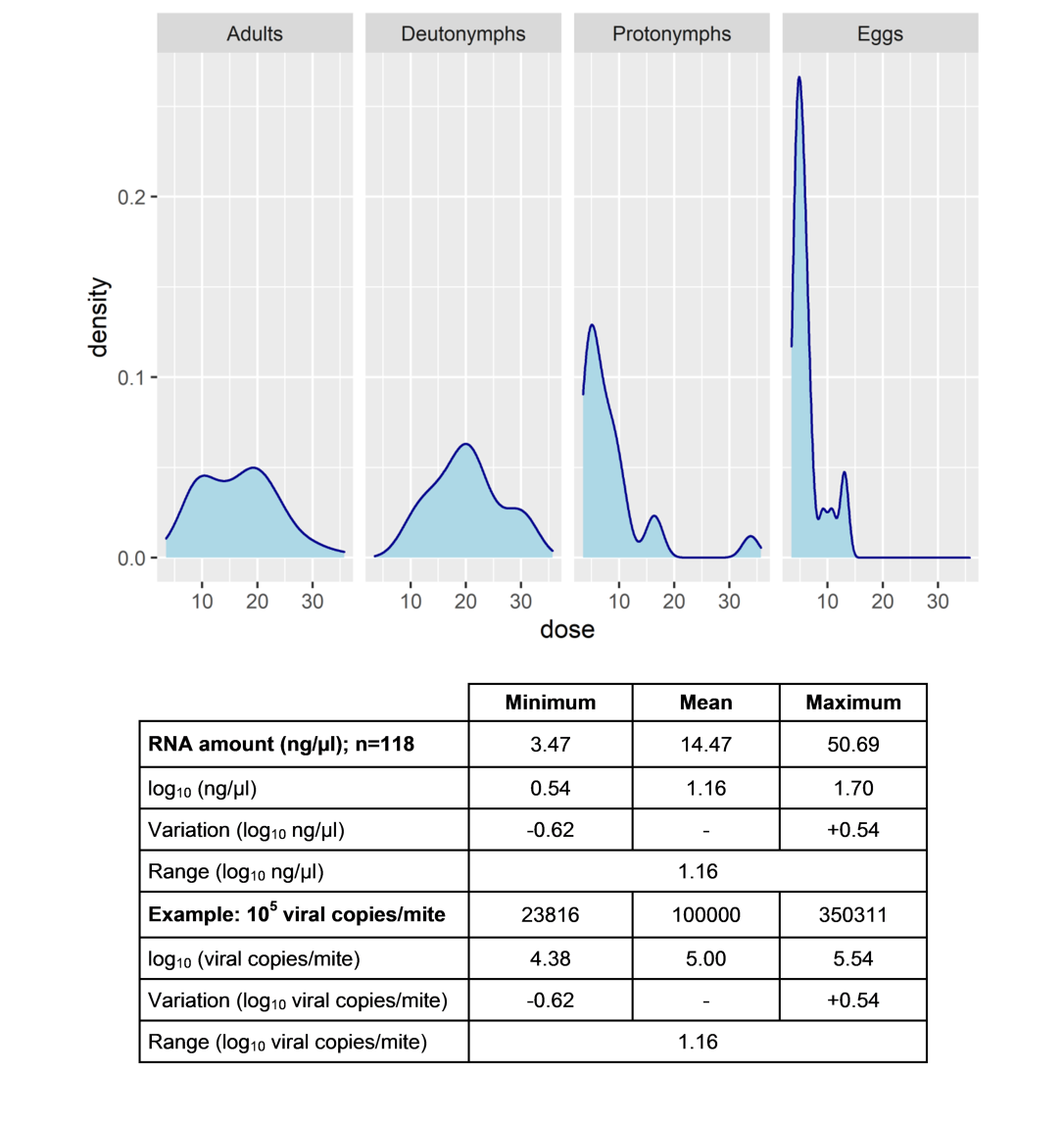
**

**Additional file 1: Fig. S1.** Distributions of *V. destructor* RNA amounts recovered after extraction, in relation to the stage of the mite considered. These variations in the total RNA amount were not taken into account because they had a limited impact on the general reproducibility of viral copy numbers quantified by RT-qPCR. Based on the ratios of RNA recovery in the 118 samples, the maximal variability of the data was estimated to be 0.62 log_10_ viral copy numbers per mite. The table provides an example with a mean copy number of 10^5^ viral copies per mite and the variations were calculated according to the convertion factor from viral copies per PCR to viral copies per mite. This variation in viral copy number remained within the tolerance limits of ± 1 log_10_ viral copies per mite.

**A B**

**
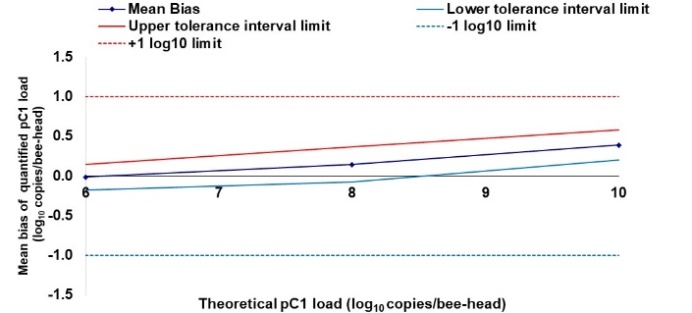

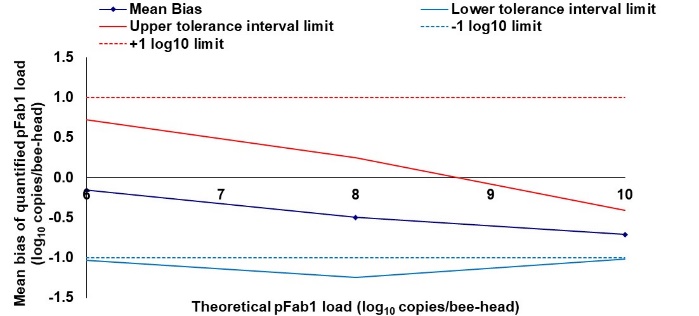
**

**Additional file 1: Fig. S2.** Accuracy profiles of the DWV-A and DWV-B quantitation methods. The assessment of the accuracy of the viral copy numbers quantified in individual bee-heads by DWV-A and DWV-B RT qPCRs was based on the construction and interpretation of accuracy profiles [17]. Crushed bee-head samples were spiked with pC1 and pFab1 recombinant plasmids including DWV-A or DWV-B VP3 coding sequence, respectively (recombinant plasmids used also for standard curves according to Schurr et al. [62], and processed for viral quantitation. Three plasmid load levels (10^6^, 10^8^ and 10^10^ copies/bee or head) were used to determine the standard deviation of reproducibility (SD_R_), the tolerance interval (± 2xSD_R_), and the mean bias between the theoretical value and the mean of the obtained values of six results per load level. Both RT-qPCR methods quantified the recombinant plasmids within the limits of ± 1.0 log_10_ copies per bee-head. The accuracy of DWV-B RT-qPCR was the lowest using spiked bee-head samples with 10^8^ plasmid copies/bee-head. **A** and **B,** Quantification accuracy of DWV-A and DWV-B RT-qPCR methods, respectively.


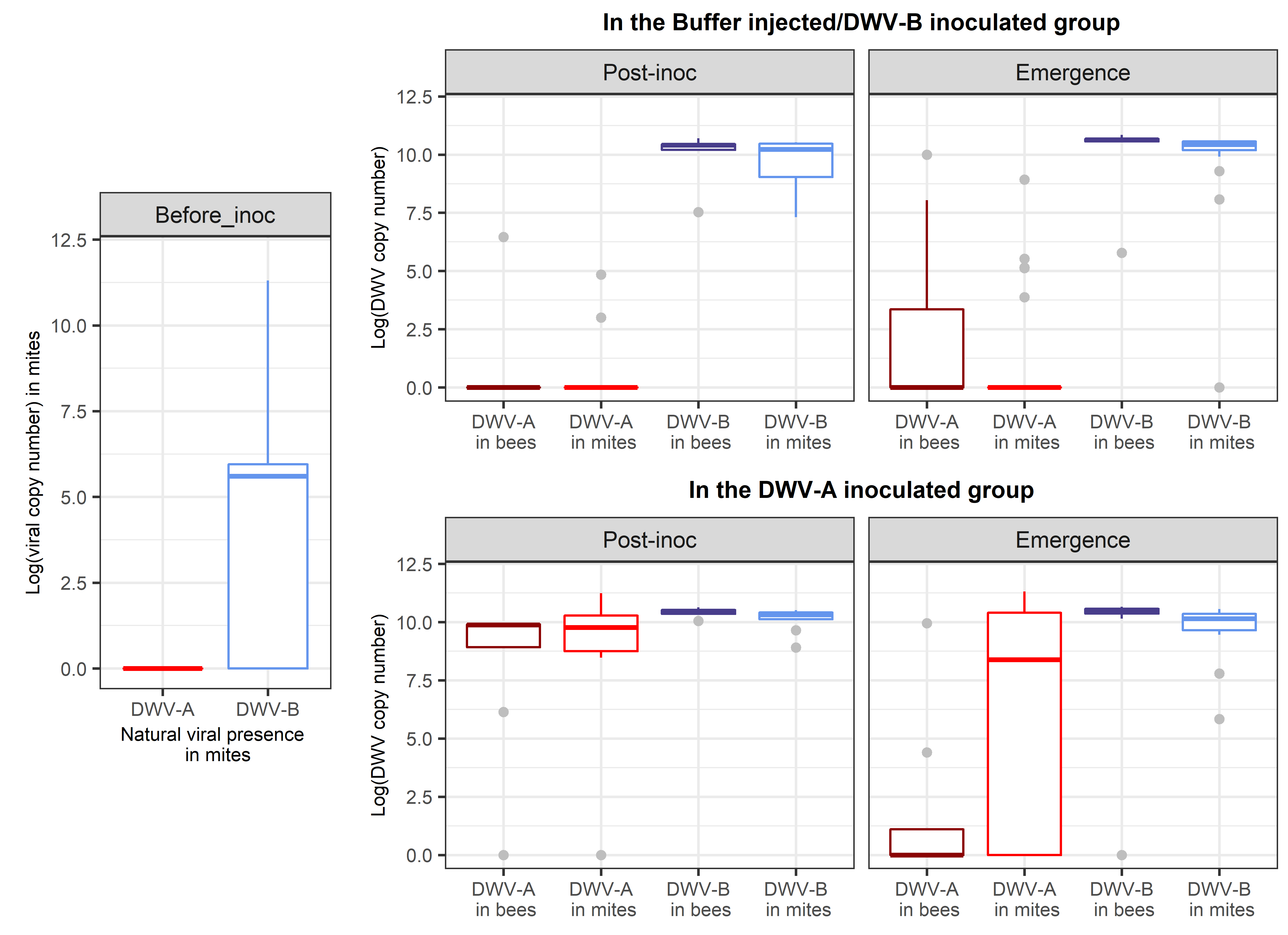


**Additional file 1: Fig. S3**. Raw viral copy numbers of *V. destructor* females from our *in vitro* study. Viral copy numbers were quantified in 9 mites from a naturally infested colony before inoculation. The mites were then transferred on artificially inoculated bee pupae for 4 days (post-inoc). The remaining mites were transferred onto untreated spinning larvae for 12 days until emergence of the bees (emergence). Bees and mites were maintained in an incubator (70% RH, 34.5°C).


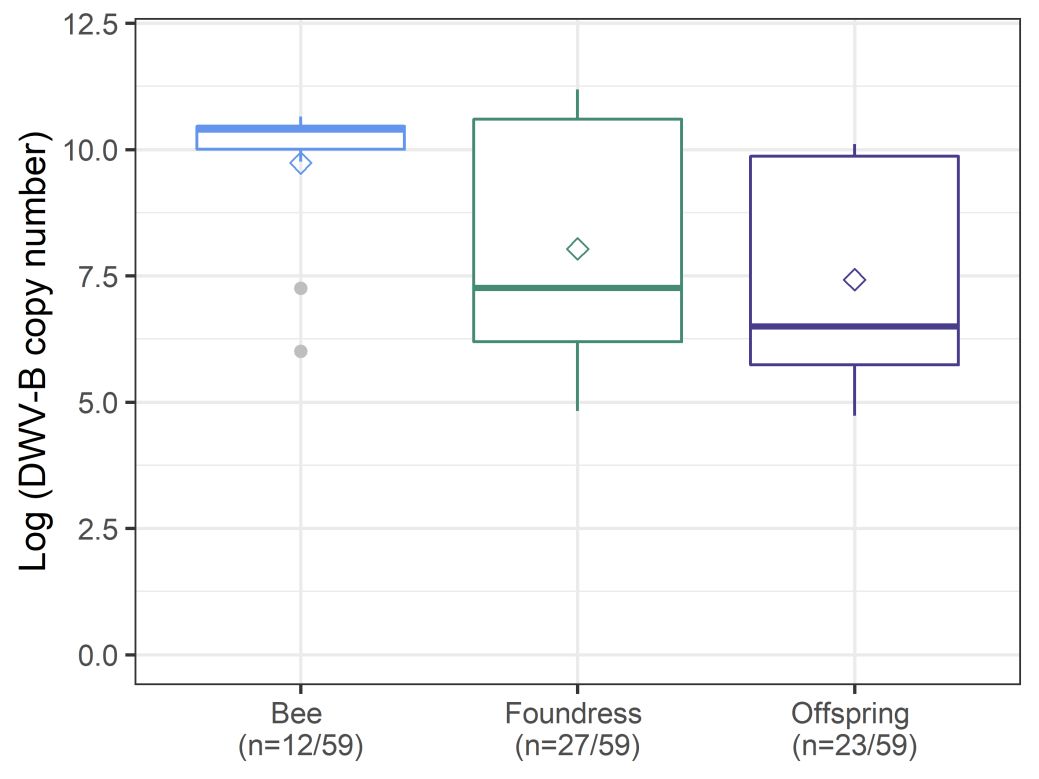


**Additional file 1: Fig. S4.** Boxplot showing the log_10_ transformed deformed wing virus-variant B (DWV-B) copy number in naturally infected bees, mite foundresses and mite offspring from the in situ study. The proportion of naturally infected individuals is shown on the x-axis. The diamonds indicate the mean value of the log_10_ transformed viral copy numbers.
